# Relationship between the Pre-ECMO and ECMO Time and Survival of Severe COVID-19 Patients: A Systematic Review and Meta-Analysis

**DOI:** 10.3390/jcm13030868

**Published:** 2024-02-01

**Authors:** Ziqi Tan, Longxiang Su, Xiangyu Chen, Huaiwu He, Yun Long

**Affiliations:** Department of Critical Care Medicine, Peking Union Medical College Hospital, Peking Union Medical College, Chinese Academy of Medical Science, Beijing 100730, China; b2023001122@pumc.edu.cn (Z.T.); sulongxiang@vip.163.com (L.S.); pumc_2022chenxiangyu@student.pumc.edu.cn (X.C.); tjmuhhw@126.com (H.H.)

**Keywords:** COVID-19, pre-ECMO time, ECMO duration, survival

## Abstract

Background: Coronavirus disease 2019 (COVID-19) is the etiology of acute respiratory distress syndrome (ARDS). Extracorporeal membrane oxygenation (ECMO) is used to support gas exchange in patients who have failed conventional mechanical ventilation. However, there is no clear consensus on the timing of ECMO use in severe COVID-19 patients. Objective: The aim of this study is to compare the differences in pre-ECMO time and ECMO duration between COVID-19 survivors and non-survivors and to explore the association between them. Methods: PubMed, the Cochrane Library, Embase, and other sources were searched until 21 October 2022. Studies reporting the relationship between ECMO-related time and COVID-19 survival were included. All available data were pooled using random-effects methods. Linear regression analysis was used to determine the correlation between pre-ECMO time and ECMO duration. The meta-analysis was registered with PROSPERO under registration number CRD42023403236. Results: Out of the initial 2473 citations, we analyzed 318 full-text articles, and 54 studies were included, involving 13,691 patients. There were significant differences between survivors and non-survivors in the time from COVID-19 diagnosis (standardized mean difference (SMD) = −0.41, 95% confidence interval (CI): [−0.53, −0.29], *p* < 0.00001), hospital (SMD = −0.53, 95% CI: [−0.97, −0.09], *p* = 0.02) and intensive care unit (ICU) admission (SMD = −0.28, 95% CI: [−0.49, −0.08], *p* = 0.007), intubation or mechanical ventilation to ECMO (SMD = −0.21, 95% CI: [−0.32, −0.09], *p* = 0.0003) and ECMO duration (SMD = −0.18, 95% CI: [−0.30, −0.06], *p* = 0.003). There was no statistical association between a longer time from symptom onset to ECMO (hazard ratio (HR) = 1.05, 95% CI: [0.99, 1.12], *p* = 0.11) or time from intubation or mechanical ventilation (MV) and the risk of mortality (highest vs. lowest time groups odds ratio (OR) = 1.18, 95% CI: [0.78, 1.78], *p* = 0.42; per one-day increase OR = 1.14, 95% CI: [0.86, 1.52], *p* = 0.36; HR = 0.99, 95% CI: [0.95, 1.02], *p* = 0.39). There was no linear relationship between pre-ECMO time and ECMO duration. Conclusion: There are differences in pre-ECMO time between COVID-19 survivors and non-survivors, and there is insufficient evidence to conclude that longer pre-ECMO time is responsible for reduced survival in COVID-19 patients. ECMO duration differed between survivors and non-survivors, and the timing of pre-ECMO does not have an impact on ECMO duration. Further studies are needed to explore the association between pre-ECMO and ECMO time in the survival of COVID-19 patients.

## 1. Introduction

Coronavirus disease 2019 (COVID-19) is a novel form of pneumonia caused by a severe acute respiratory syndrome coronavirus 2 (SARS-CoV-2) infection. According to the World Health Organization (WHO), globally, as of 21 February 2023, there have been 757,264,511 confirmed cases of COVID-19, including 6,850,594 deaths (URL: https://covid19.who.int/, accessed on 21 February 2023). While most COVID-19 patients experience mild symptoms, several studies suggest that the mortality rate of COVID-19 is still as high as 53–67% [1,2]. In China, the overall case-fatality rate for COVID-19 is 2.3% (1023 deaths out of 44,672 confirmed cases), with approximately 2087 patients in critical condition accounting for 5% of confirmed cases. Among critically ill COVID-19 patients, there were approximately 1023 deaths, resulting in a mortality rate of 49% [3]. Based on the experience of the previous viral outbreak period [4,5], extracorporeal membrane oxygenation (ECMO) has been identified as an important form of life support for critically ill patients. It can be effective in treating severe respiratory failure due to acute respiratory distress syndrome (ARDS) and supporting gas exchange in patients who have failed conventional mechanical ventilation (MV). At the same time, Extracorporeal Life Support Organization (ELSO) guidelines suggest that ECMO can be used as a rescue treatment for critically ill COVID-19 patients who do not respond to conventional ARDS therapy [6].

Despite optimism regarding the potential role of ECMO in COVID-19 treatment, several studies still report a high mortality rate. A meta-analysis found a mortality rate of 37% in patients who received ECMO for COVID-19 in 2020 [7]. Additionally, ELSO registry data reported an increase in mortality rates for the use of ECMO in COVID-19 patients, rising from 37% at the beginning of 2020 to 52% at the end of the year [8,9]. Late initiation of ECMO may be an independent risk factor for increased mortality. Li et al. revealed that early initiation of ECMO was associated with decreased 60-day mortality after ECMO (50% vs. 88%, *p* = 0.044) [10]. However, Mathilde et al. reported that late ECMO treatment in patients with refractory ARDS related to SARS-CoV-2 does not seem to be associated with an excess risk of mortality [11]. The optimal timing for initiating ECMO in COVID-19 treatment is currently uncertain and controversial. There is insufficient global evidence to assess the effectiveness of ECMO timing, and no studies have shown whether ECMO duration affects mortality in severe COVID-19 patients.

To aid clinicians in accurately determining the timing of ECMO use and to improve the use and management of ECMO in severe COVID-19 patients, we conducted a systematic review and meta-analysis. This study focuses on the timing of pre-ECMO and ECMO duration in COVID-19 patients, to clarify the effects of pre-ECMO and ECMO duration on COVID-19 patient survival, and to guide current clinical practice and future research. Furthermore, we have further investigated the linear correlation between pre-ECMO time and ECMO duration.

## 2. Methods

The protocol was registered in PROSPERO (International Prospective Register of Systematic Reviews URL: https://www.york.ac.uk/crd/, accessed on 3 March 2023), with the registration number CRD42023403236. This meta-analysis was conducted according to the guidelines of the Preferred Reporting Item for Systematic Review and Meta-Analysis 2020 (PRISMA 2020) (Supplementary Table S1).

### 2.1. Literature Search

Three databases (PubMed, Embase, and the Cochrane Library databases) were used as our search libraries. Other sources, such as the Critical Care Medicine website (URL: http://www.ccmjournal.com, accessed on 21 October 2022), the Critical Care website (URL: http://ccforum.com, accessed on 21 October 2022), and the American Journal of Respiratory and Critical Care Medicine website (URL: http://ajrccm.atsjournals.org, accessed on 21 October 2022), were also searched. Without language restriction, the following search Medical Subject Headings (MeSHs) were used to retrieve advanced articles from inception to 21 October 2022 according to the PICOS (population, intervention/exposure, comparison, outcome, and study design) principle: (1) for patients: “COVID-19” OR “SARS-CoV-2 Infection”; (2) for intervention and comparison: “Extracorporeal Membrane Oxygenations”; (3) for outcome: “Outcome” OR “Survival”. Supplementary Table S2 describes the detailed search strategy.

### 2.2. Study Selection

Reference management software, Endnote X9.3.3 software (Thomson Reuters, New York, NY, USA), was used to organize all studies. All titles and abstracts were reviewed after removing duplicates. Then, the full-text assessment was performed following an initial screening to consider eligibility for inclusion.

According to the PICOS principle, the inclusion criteria were as follows: (1) for population: adults (aged > 18 years) who were diagnosed with COVID-19 infection by a positive real-time reverse transcriptase-polymerase chain reaction (RT-PCR) assay and who underwent ECMO for hypoxemia; (2) for intervention, comparison, and outcome: studies on the association between differences in the duration of ECMO and patient survival, reporting corresponding risk estimates, such as odds ratios (ORs), relative risks (RRs), or hazard ratios (HRs), and their corresponding 95% confidence intervals (CIs), or providing related data; (3) for study design: random controlled trials (RCTs), post hoc analyses of RCTs, observational cohort studies, and cross-sectional studies.

Our exclusion criteria included: (1) reviews and studies with insufficient data; (2) populations with other established conditions (e.g., diabetes population) at baseline; and (3) if the same population was used in multiple studies, we excluded the less informative article.

### 2.3. Data Extraction and Quality Assessment

Data extraction and quality assessment of the included studies were conducted independently by two researchers. Information extracted included author, year of publication, country, study design, data source, follow-up time, sample size, mean age, gender, ECMO type, ECMO initiation, baseline comorbidities, other treatment, time period category, baseline data, estimated effect, and adjustments.

The Newcastle–Ottawa Scale (NOS) was used to assess the quality of the included observational studies. The scores range from zero to nine to evaluate the selection, comparability, and outcome of articles. Studies with NOS scores of one to three, four to six, and seven to nine are considered to be of low, medium, and high quality, respectively [12,13].

### 2.4. Statistical Analysis

All data (e.g., age, time) expressed as quartiles and medians were converted to the mean and standard deviation [14,15,16]. To elucidate the differences in baseline pre-ECMO and ECMO time between survivors and non-survivors in severe COVID-19 patients, we respectively pooled the pre-ECMO (time from symptom onset to ECMO, time from COVID-19 diagnosis to ECMO, time from hospital admission to ECMO, time from intensive care unit (ICU) admission to ECMO, and time from MV or intubation to ECMO) and ECMO time of survivors and non-survivors using the inverse-variance method and random model to generate the effect size standardized mean difference (SMD).

OR is approximately equivalent to RR or HR when the outcome is rare [17]. Therefore, to determine the relationship between time and survival, the ORs, RRs, and HRs and their 95% CIs were pooled, respectively, using a random-effects model to improve reliability. We estimated the effect size by calculating the natural logarithm of the OR, RR, or HR (log [OR], log [RR], or log [HR]) and their standard error (SElog [OR], SElog [RR], or SElog [HR]) to be pooled. Pre-ECMO and ECMO time were analyzed as a categorical variable, with the group with the longest time compared to the group with the shortest time. Time was analyzed as a continuous variable, and the units of the time (per one-day increase) were standardized.

A linear regression model was used to identify directional associations between ECMO duration and pre-ECMO time, including time from symptom onset to ECMO, time from COVID-19 diagnosis to ECMO, time from hospital admission to ECMO, time from ICU admission to ECMO, and time from MV or intubation to ECMO.

SPSS version 16.0 software (SPSS Inc., Chicago, IL, USA) and Review Manager (RevMan) version 5.4 (The Cochrane Collaboration 2014; Nordic Cochrane Center Copenhagen, Denmark) were used for statistics and analysis. A *p*-value of < 0.05 was considered statistically significant.

### 2.5. Heterogeneity Test, Publication Bias, and Sensitivity Analysis

We calculated the statistical *p*-value using the Q-test, with a *p*-value < 0.1 representing a significant difference between the two groups. To estimate the degree of heterogeneity, we applied the I^2^ test between studies. Low heterogeneity, moderate heterogeneity, and high heterogeneity were defined as I^2^ < 50%, 50–75%, and >75%, respectively [18]. Sensitivity analyses were performed by omitting each study in turn.

## 3. Results

### 3.1. Literature Search

**Figure 1:**
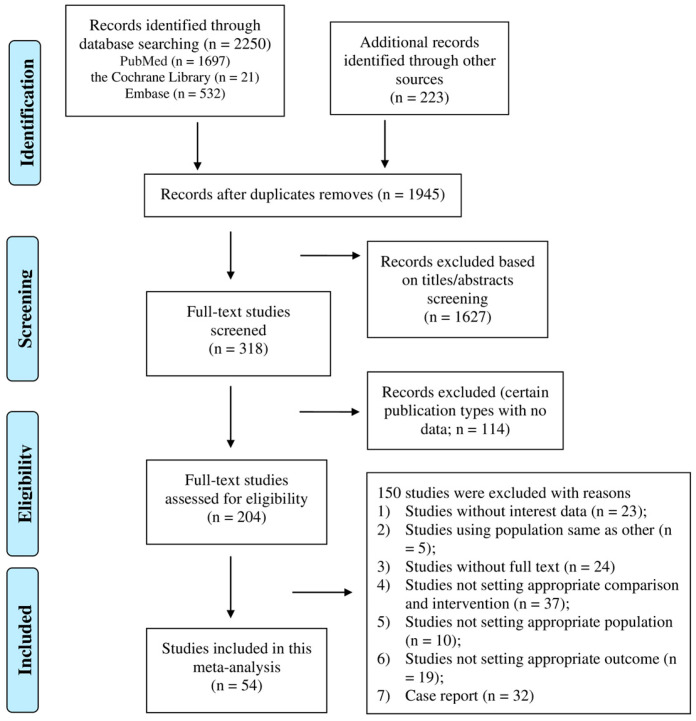
Flow chart of study selection in the systematic review and meta-analysis of ECMO time difference in the survival of COVID-19.

Figure 1 shows the flowchart of the database search process. According to the preformulated search protocol, a total of 2473 publications were identified in the initial search (PubMed = 1697; Cochrane Library = 21; Embase = 532; other sources = 223). After excluding 528 duplicates and 1627 irrelevant publications after title and abstract screening, 318 articles were processed for full-text assessment. After removing 114 articles with specific publication types without data, a further 150 articles were excluded for the following reasons: (1) studies without data of interest (*n* = 23); (2) duplicated cohorts (*n* = 5); (3) studies without full text (*n* = 24); (4) studies not focusing on pre-ECMO or ECMO time (*n* = 37); (5) studies not focusing on specific populations (*n* = 10); (6) studies not focusing on target outcome (*n* = 19); and (7) case reports (*n* = 32). All excluded studies (*n* = 150) and their corresponding reasons are listed in Supplementary Table S3. Ultimately, our meta-analysis included a total of 54 studies with 55 cohorts [19,20,21,22,23,24,25,26,27,28,29,30,31,32,33,34,35,36,37,38,39,40,41,42,43,44,45,46,47,48,49,50,51,52,53,54,55,56,57,58,59,60,61,62,63,64,65,66,67,68,69,70,71,72].

Other sources include the Critical Care Medicine website, the Critical Care website, and the American Journal of Respiratory and Critical Care Medicine website.

### 3.2. Study Characteristics

The basic characteristics of all included studies are shown in Table 1. Fifty-four cohort studies [19,20,21,22,23,24,25,26,27,28,29,30,31,32,33,34,35,36,37,38,39,40,41,42,43,44,45,46,47,48,49,50,51,52,53,54,55,56,57,58,59,60,61,62,63,64,65,66,67,68,69,70,71,72] were published from 2020 to 2022, including 13,691 severe COVID-19 patients on ECMO, with a mean age ranging from 44 to 67 years. The sample size ranged from 11 to 7135, and the duration of follow-up from ECMO decannulation to two years. Of these articles, seventeen cohorts reported the time from symptom onset to ECMO [22,24,26,31,39,40,42,44,50,54,55,56,61,63,65,67,70], four reported the time from COVID-19 diagnosis to ECMO [34,38,47,52], eleven reported the time from hospital admission to ECMO [20,24,25,39,42,46,48,61,64,66,71], four reported time from ICU admission to ECMO [23,29,44,61], thirty-nine reported time from intubation or MV to ECMO [19,24,26,27,28,29,30,33,34,35,36,37,38,39,40,42,43,44,45,47,50,51,52,53,54,55,56,57,58,59,61,62,64,66,67,68,70,71,72], thirty-five reported duration of ECMO [19,20,21,23,24,27,28,29,32,33,34,35,36,37,38,39,41,42,43,44,45,46,47,48,49,50,55,58,61,63,66,69,70,71,72]. Of the total articles, twenty-four studies were conducted in the Americas (twenty-two in the United States of America (USA) [19,20,21,24,25,32,34,35,38,39,46,47,52,54,55,57,58,61,65,66,68,70], one in Argentina [26], one in Chile [29]), twenty-three in Europe (nine in France [22,29,33,42,44,49,50,59,60], six in Germany [31,37,41,48,62,71], two in the United Kingdom of Great Britain and Northern Ireland (UK) [27,72], two in Spain [51,56], one in Finland [23], one in Austria [36], one in Poland [64], one in Italy [45]), seven in Asia (three in China [28,40,67], two in Japan [63,69], one in Republic of Korea [43], one in Saudi Arabia [53]). In addition, forty-two of them were retrospective cohort studies [19,20,21,23,24,26,27,30,31,32,35,36,37,39,40,41,42,43,44,45,46,47,48,50,52,53,54,57,58,59,60,61,62,63,64,65,67,68,69,70,71,72], ten were prospective cohort studies [22,25,28,33,34,38,49,51,55,66], and two were ambispective cohort studies [29,56].

### 3.3. Quality Evaluation

Of the fifty-four included articles, five studies had an NOS score of five [51,63,65,68,69], and twenty-three had a score of six [20,24,25,26,27,32,35,38,40,43,44,45,46,47,48,50,52,64,66,67,70,71,72]. Effect sizes and follow-up times were not reported, raising concerns about selection and outcome bias. The remaining studies were all of high quality, with a score of more than six (Supplementary Table S4).

### 3.4. Baseline Differences and Meta-Analysis of Pre-ECMO Time in COVID-19 Patients’ Survival

#### 3.4.1. Time from Symptom Onset to ECMO

**Figure 2:**
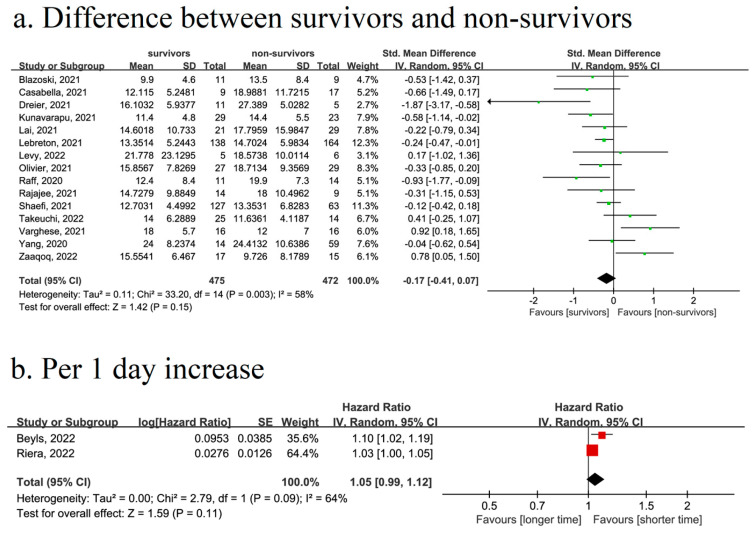
Forest plot for the association between time from symptom onset to ECMO and survival in COVID-19 patients. (**a**). Forest plot showing the time differences between survivors and non-survivors in COVID-19 patients. (**b**). Forest plot for the association between time and survival, analyzed as continuous variables (per one-day increase).

Fifteen articles with 475 survivors and 472 non-survivors showed differences in the time from symptom onset to ECMO [4,24,26,31,39,40,42,50,54,55,61,63,65,67,70]. There was no time difference between survivors and non-survivors (SMD = −0.17, 95% CI: [−0.41, 0.07], *p* = 0.15; I^2^ = 58%, *p* = 0.003) (Figure 2a).

Two cohort studies, including 373 COVID-19 patients, were used for the analysis of time from symptom onset to ECMO and survival [22,56]. The results showed that there was no significant association between a longer time and the risk of death (HR = 1.05, 95% CI: [0.99, 1.12], *p* = 0.11; I^2^ = 64%, *p* = 0.09) (Figure 2b).

#### 3.4.2. Time from COVID-19 Diagnosis to ECMO

Four cohorts, including 480 survivors and 660 non-survivors, showed the difference in time from COVID-19 diagnosis to ECMO [34,38,47,52]. The result shows that non-survivors had a longer time than survivors (SMD = −0.41, 95% CI: [−0.53, −0.29], *p* < 0.00001), with low heterogeneity (I^2^ = 0%, *p* = 0.39) (Supplementary Figure S1A).

#### 3.4.3. Time from Hospital Admission to ECMO

Analyzing eleven studies (495 survivors and 510 non-survivors) [20,24,25,39,42,46,48,61,64,66,71], non-survivors had a longer time from hospital admission to ECMO than survivors (SMD = −0.53, 95% CI: [−0.97, −0.09], *p* = 0.02), with high evidence of heterogeneity (I^2^ = 88%, *p* < 0.00001) (Supplementary Figure S1B).

#### 3.4.4. Time from ICU Admission to ECMO

Four cohorts with 233 survivors and 176 non-survivors reported the difference in baseline time from ICU admission to ECMO between the two groups [23,29,44,61]. Compared with survivors, non-survivors had a longer time (SMD = −0.28, 95% CI: [−0.49, −0.08], *p* = 0.007; I^2^ = 3%, *p* = 0.38) (Supplementary Figure S1C).

In addition, Daviet’s study showed that there was no association between time from ICU admission to ECMO and survival in COVID-19 patients (OR = 1.175, 95% CI: [0.984, 1.403], *p* = 0.075) [29]. Instead, Raff’s team and Schmidt’s team reported that each additional day spent in the ICU prior to ECMO cannulation conferred an adjusted RR of death of 1.04 (95% CI: [1.01, 1.09], *p* = 0.027) [54], and a longer interval from ICU admission to ECMO tended to be associated with higher 90-day mortality (more than ten days: less than four days HR = 3.02, 95% CI: [1.15, 7.92], *p* = 0.066), respectively.

#### 3.4.5. Time from Intubation or MV to ECMO

Thirty-four cohorts, including 1700 survivors and 1874 non-survivors, showed that the time from intubation or MV to ECMO differed between survivors and non-survivors [19,24,26,27,28,29,30,33,34,35,36,38,39,40,42,43,44,45,47,50,52,54,55,57,59,61,62,64,66,67,68,70,71,72]. Non-survivors had a longer time from intubation or MV to ECMO than survivors (SMD = −0.21. 95% CI: [−0.32, −0.09], *p* = 0.0003; I^2^ = 53%, *p* = 0.0002) (Supplementary Figure S1D(a)).

Ten studies were pooled to explore the association between time from intubation or MV to ECMO and survival, respectively [30,34,36,37,39,42,51,53,56,58]. Pooled results showed that there was no association between a shorter time and an increased survival in analyses of the highest versus lowest time groups (OR = 1.18, 95% CI: [0.78, 1.78], *p* = 0.42; I^2^ = 39%, *p* = 0.20) (Supplementary Figure S1D(b)) or per one-day increase (OR = 1.14, 95% CI: [0.86, 1.52], *p* = 0.92; I^2^ = 90%, *p* < 0.0001; HR = 0.99, 95% CI: [0.95, 1.02], *p* = 0.39; I^2^ = 70%, *p* = 0.02) (Supplementary Figure S1D(c)). Excluding the Lebreton study [42], we reduced the I^2^ from 90% to 0%, and the OR became 1.31 (95% CI: [1.14, 1.51], *p* = 1.00).

### 3.5. Baseline Differences in ECMO Duration in COVID-19 Patients’ Survival

**Figure 3:**
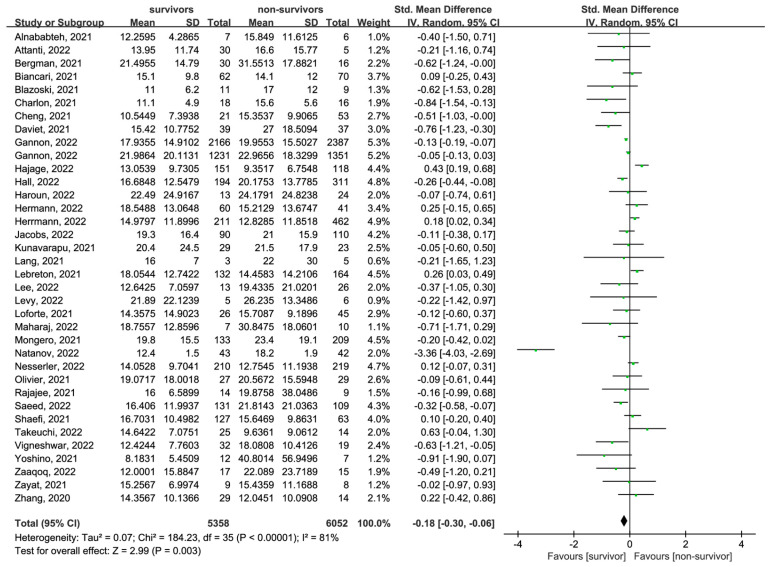
Forest plot showing the differences in ECMO duration between survivors and non-survivors in COVID-19 patients. Abbreviations: COVID-19: Coronavirus disease 2019; ECMO: extracorporeal membrane oxygenation; ICU: intensive care unit; MV: mechanical ventilation.

Thirty-five articles with thirty-six cohorts (5358 survivors and 6052 non-survivors) reported the difference in ECMO duration between survivors and non-survivors [19,20,21,23,24,27,28,29,32,33,34,35,36,37,38,39,41,42,43,44,45,46,47,48,49,50,55,57,61,63,66,69,70,71,72]. The result showed that non-survivors had a longer ECMO duration compared to survivors (SMD = −0.18, 95% CI: [−0.30, −0.06], *p* = 0.003; I^2^ = 81%, *p* < 0.00001) (Figure 3).

### 3.6. Regression Analysis of Pre-ECMO Time and ECMO Duration

Twenty-nine studies were involved in the regression analysis [19,20,23,24,27,28,29,33,34,35,36,38,39,42,43,44,45,46,47,48,50,55,58,61,63,65,70,71,72]. The results of the linear regression analysis indicate that the time before ECMO was significantly associated with the duration of ECMO, either in survivors (time from symptom onset to ECMO β = 0.35, 95% CI: [−0.49, 1.19], *p* = 0.37; time from COVID-19 diagnosis to ECMO β = 0.01, 95% CI: [−19.57, 19.60], *p* = 0.99; time from hospital admission to ECMO β = −0.92, 95% CI: [−1.85, 0.01], *p* = 0.051; time from ICU admission to ECMO β = 0.46, 95% CI: [−0.43, 1.34], *p* = 0.16; time from intubation or MV to ECMO β = −0.16, 95% CI: [−0.87, 0.55], *p* = 0.65) or in non-survivors (time from symptom onset to ECMO β = 0.67, 95% CI: [−0.42, 1.76], *p* = 0.19; time from COVID-19 diagnosis to ECMO β = −0.29, 95% CI: [−28.26, 27.67], *p* = 0.92; time from hospital admission to ECMO β = 0.27, 95% CI: [−1.13, 1.68], *p* = 0.66; time from ICU admission to ECMO β = 1.68, 95% CI: [−0.38, 3.74], *p* = 0.07; time from intubation or MV to ECMO β = 0.09, 95% CI: [−0.70, 0.88], *p* = 0.82) (Supplementary Figure S2).

### 3.7. Sensitivity Analysis, Subgroup Analysis, and Publication Bias

We performed a sensitivity analysis for the time from intubation (MV) to ECMO. In the analysis of the OR per one-day increase group, by excluding Lebreton’s study, the OR became 1.31 (95% CI: 1.14–1.51). Other sensitivity analyses by deleting one-by-one studies showed consistent results (Supplementary Figure S3). Due to the limited number of included studies (*n* < 10), subgroup and publication bias analyses were not performed according to the guidelines and predefined criteria.

## 4. Discussion

### 4.1. Main Finding

Based on the meta-analysis of 54 studies with 55 cohorts and 13,691 COVID-19 patients, it was found that: (1) non-survival ECMO patients had a longer pre-ECMO time than survivors, including time from COVID-19 diagnosis to ECMO, time from hospital admission to ECMO, time from ICU admission to ECMO, time from intubation or MV use to ECMO, and there was no sufficient evidence to prove the association between pre-ECMO time and COVID-19 survival; (2) there is a longer ECMO time in non-survival COVID-19 patients than survivors; (3) there is no linear relationship between pre-ECMO time and ECMO duration. Although our analysis showed differences in pre-ECMO and ECMO time for survivors versus non-survivors, the relationship between the two needs to be further explored.

The impact of ECMO on the COVID-19 prognosis is significant. The timing and duration of ECMO are significant factors to consider when treating critically ill patients with COVID-19. Previous studies have confirmed that early ECMO intervention after MV improves survival in patients with ARDS caused by influenza A virus subtype H1N1 pneumonia [73]. Most patients with ARDS and severe SARS-CoV-2 pneumonia receive delayed treatment and deteriorate rapidly. Almost all of the studies we included indicated that the pre-ECMO period was shorter in the survival group than in the non-survival group for COVID-19 patients treated with ECMO. Li et al. demonstrated that patients with COVID-19 who received early ECMO treatment had lower mortality than those who received late ECMO treatment [10]. Furthermore, it has been suggested that early use of ECMO may lead to a better prognosis [74], while prolonged ECMO treatment may increase the risk of death and multi-organ failure [75]. A study has shown that an invasive MV duration longer than 7 days before ECMO is a significant prognostic factor for death [76]. Therefore, it is recommended to initiate ECMO as soon as possible [77].

The management of ECMO is also a major factor in the mortality of severe COVID-19 patients. ECMO is classified into three categories based on the route of blood transfusion: veno-venous ECMO (VV-ECMO), venous-arterial ECMO (VA-ECMO), and hybrid ECMO configurations. VV-ECMO provides only respiratory assistance, while VA-ECMO provides both circulatory and respiratory assistance. The choice of ECMO category may impact the patient’s prognosis. In critically ill COVID-19 patients, VV-ECMO is the option when circulatory failure is not present. When circulatory failure is present, such as in the case of refractory hypoxemia associated with ARDS or shock associated with septic cardiomyopathy, VA-ECMO, or hybrid ECMO, is required. Because the use of different ECMOs is not reported in detail in the included literature, we did not perform subgroup analyses for this classification. A meta-analysis was conducted to investigate the effect of exposure to severe hyperoxemia on mortality and neurological outcomes in VA-ECMO-supported patients. The findings showed that exposure to severe hyperoxemia is associated with higher mortality (OR = 1.80, 95% CI: 1.16–2.78) and a poorer neurological outcome (OR = 1.97, 95% CI: 1.30–2.9). Therefore, it is recommended that efforts be made to avoid severe hyperoxemia during VA-ECMO support [78]. In addition, the prolonged use of ECMO increases the chances of nosocomial infections due to COVID-19 infection, which leads to impaired immune function in patients [79,80]. Therefore, it is crucial to improve intubation management. Also, ECMO anticoagulation management needs to pay close attention. Several anticoagulation strategies have been implemented to improve the outcome of COVID-19 patients treated with ECMO. For instance, nafamostat mesylate, a promising anticoagulant drug, could be used for systemic anticoagulation during ECMO administration. It may be able to serve as a feasible and safe option for anticoagulation during ECMO in critically ill patients with COVID-19 [81]. Coagulation tests, such as activated clotting time, should be monitored regularly by healthcare professionals to avoid thrombosis or bleeding [82]. In addition to this, the COVID-19 pandemic has severely strained intensive care resources in hospitals [83]. Although patients may meet the ECMO to Rescue Lung Injury in Severe ARDS trial (EOLIA trial) criteria, ECMO support may not be initiated in time [84,85].

Studies investigating the association between pre-ECMO and ECMO duration and survival in COVID-19 patients have produced inconsistent results. After multifactorial adjustment, Nesserler et al. reported a higher mortality rate with longer pre-ECMO duration (HR = 1.74, 95% CI: 1.07–2.83) [49], while Saeed et al. did not reach the same conclusion (HR = 1.01, 95% CI: 0.98–1.03) [57]. Our results indicated a difference in pre-ECMO time (e.g., MV or time to intubation to ECMO) between COVID-19 survivors and non-survivors, but this time is not statistically related to COVID-19 survival. It is important to note that the limited number of studies may have biased these results. Therefore, caution is advised when interpreting these findings, and further studies are needed to validate the relationship between ECMO-related time and the survival of critically ill COVID-19 patients. In addition, the inconsistency of the two statistical methods introduced some bias. As we are unable to adjust for all confounding factors, only survival and non-survival were considered when combining for time. Instead, in conducting research on the relationship between them, multifactor-adjusted studies were included, in which they adjusted for confounders such as age, comorbidity, type of ECMO, Respiratory ECMO Survival Prediction (RESP) score, and sequential organ failure assessment (SOFA) score, which was the main reason for the inconsistent results. In addition, we included studies for both univariate and multivariate analyses, and due to the limited number of included articles, we were unable to separately analyze studies adjusted for confounding factors. The effect of confounding factors on outcomes still needs to be elucidated.

It is also worth discussing whether the timing of pre-ECMO has an impact on the timing of ECMO. According to our results, in either survivors or non-survivors, pre-ECMO time showed no linear relationship with ECMO duration. In combination with previous relevant clinical studies and the recommendations of the ELSO, the timing of ECMO should be considered when the patient is at or above 50% risk of death in reference to any cause of hypoxic respiratory failure, and ECMO treatment should be initiated when the patient is at or above 80% risk of death. Currently, ECMO is only used as a supportive tool to allow time for primary disease treatment, rather than as a treatment in itself. Early use of ECMO can prevent cellular damage to organs and tissues caused by hypoxic metabolism and provide a favorable opportunity to treat the primary disease. Therefore, the duration of ECMO use is closely related to the improvement of the primary disease. That is, the duration of ECMO use may be shortened if earlier use of ECMO provides more adequate time to better support the treatment of the primary morbidity and if the primary morbidity improves during ECMO use. In contrast, a shorter duration of pre-ECMO does not mean a shorter duration of ECMO if the primary morbidity is not controlled. Although the early use of ECMO in severe COVID-19 patients is supported, there are no more studies that clearly show a relationship between pre-ECMO and the duration of ECMO use, and this issue still requires ongoing attention.

### 4.2. Underlying Mechanism

The impact of initiating ECMO early on patients with severe COVID-19 is multifactorial. Firstly, it is important to note that ARDS in COVID-19 patients aligns with the Berlin definition [86]. However, Gattinoni et al. proposed an alternative perspective, suggesting that lung compliance is significantly reduced in COVID-19 patients and that severe hypoxemia is more commonly associated with ventilation/perfusion (VA/Q) mismatch. For patients with COVID-19, conventional treatments such as mechanical ventilation or prone ventilation do not improve oxygenation by recruiting collapsed areas [87]. Therefore, early use of ECMO can benefit patients by minimizing ventilator-induced lung injury. Secondly, it has been found that death from COVID-19 is closely linked to hypercoagulable and thrombotic states, as supported by Yin et al. According to their report, platelet levels are higher in COVID-19 patients than in non-COVID-19 pneumonia patients [88]. When administering ECMO cannulation, a systemic anticoagulation strategy is typically employed to ensure safety [89]. Also, the ELSO guideline proposes to consider anticoagulation therapy targeting the higher end of normal for ven-venous ECMO in COVID-19 patients due to their known hypercoagulable state [6]. This approach reduces the risk of thrombosis and subsequent death resulting from the intrinsic thrombotic state, providing an additional benefit to severe COVID-19 patients. Thirdly, hospital-acquired infections are more prevalent in hospitals than in other settings. Prolonged use of ECMO is associated with an increased risk of nosocomial infections [90], which may contribute to mortality.

### 4.3. Clinical Implications

Neither the clinical guidelines related to COVID-19 published by the WHO [91] nor the regularly updated guidelines of the National Institutes of Health [92] take a positive position on whether to apply ECMO to patients with severe COVID-19. However, according to the EOLIA trial, the ELSO made a standard recommendation that ECMO therapy could be used in certain patients with COVID-19 [6]. Subsequently, the Korean Society for Thoracic and Cardiovascular Surgery (KSTCVS) [93] and Chinese experts [94,95,96] have also recommended ECMO as a salvage therapy for patients with severe COVID-19 who have not responded to conventional ARDS therapy.

Our results show a significant difference between COVID-19 survivors and non-survivors in terms of pre-ECMO time and ECMO duration. This suggests that by adjusting the timing of ECMO, there may be an impact on the survival of patients with severe COVID-19. However, our study failed to identify an association between pre-ECMO and ECMO timing and the survival of COVID-19 patients. Therefore, additional studies and more articles are required to confirm the relationship between ECMO timing and survival in patients with COVID-19, to determine the optimal timing and duration of ECMO treatment for COVID-19, and thus to improve survival in severe COVID-19 patients. In the meantime, further guideline updates or clinical trials may highlight the differences in ECMO-related time in COVID-19 patients and will still require our continued attention.

In addition, it is worth noting that prior treatment with ECMO is crucial for patients with severe COVID-19. Non-invasive respiratory support has been shown to reduce the need for intubation and invasive MV [60], but mortality may be increased in patients with COVID-19 who fail non-invasive ventilation strategies [97,98]. Indeed, dysregulated spontaneous breathing, associated with wide transpulmonary pressure swings, may increase the risk of harmful “patient spontaneous induced lung injury” on non-invasive MV or high-flow nasal cannula therapy, leading to a greater susceptibility to pneumonia and fibrosis [99,100]. Also, based on existing studies, emergency tracheal intubation and MV are required for severe COVID-19 patients exhibiting signs of respiratory distress, hypoxemia, or encephalopathy. A previous meta-analysis demonstrated that a longer duration of invasive MV was associated with a poor prognosis [101]. Patients who remain unsuccessful after optimization of MV strategies may be considered for pulmonary resuscitation strategies. While invasive MV can benefit patients, it can also cause ventilator-associated lung injury if not used properly. This can be caused by high driving pressure, which has been linked to increased mortality in severe cases of COVID-19.

A study has shown that the prone position can relieve atelectasis even at low positive end-expiratory pressure (PEEP) levels [102]. In patients with severe hypoxia, the prone position can be considered an operation to preserve PEEP. Thus, the prone-position strategy can balance the adverse effects of invasive MV [30]. At the same time, the prone position can improve oxygenation in patients with prolonged hypoxemia during ECMO. When using lung-protective ventilation to reduce lung injury, the addition of prone position therapy in conjunction with ECMO can further aid and optimize alveolar recovery. This combination of strategies (ECMO and prone position) has been shown to improve overall survival [103]. It is also important to consider potential complications when applying the combined strategy, such as accidental decannulation and kinking of the infusion system due to the prone position, as well as coagulation disorders and pressure ulcers.

### 4.4. Comparison with Prior Meta-Analysis

Previous meta-analyses have compared the effect of the presence or absence of ECMO use on COVID-19 mortality or the difference in mortality between COVID-19 and other virus-induced diseases treated with ECMO [7,77,104,105,106]. For example, Kusumawardhani’s study found a significantly higher incidence of mortality in COVID-19 patients treated with ECMO compared to those not treated with ECMO (OR = 15.79, 95% CI: 4.21–59.28, *p* < 0.0001) [106]. Ramanathan’s meta-analysis reported an in-hospital ECMO mortality rate of 37.1% for COVID-19 patients, which is similar to that of patients with non-COVID-19-related ARDS [7].

Currently, no studies have summarized the effect of pre-ECMO and ECMO duration on mortality in COVID-19 patients. Our meta-analysis is the first study based on this. We analyzed pre-ECMO and ECMO timing in COVID-19 survivors versus non-survivors, as well as the association between the ECMO-related time and COVID-19 survival. In addition, we sought to explore the relationship between pre-ECMO time and ECMO time in patients with COVID-19. Although no robust and definitive results were obtained, our study gives direction for future research.

### 4.5. Strength and Limitation

Our study is the first to investigate the relationship between ECMO duration and survival in patients with severe COVID-19. First, we conducted a comprehensive analysis of relevant literature without language restrictions, focusing on a specific and exclusive population. Second, we explored the relationship between ECMO duration and death in COVID-19 patients in two ways: by using continuous variables to explore time-specific differences and by analyzing adjusted effect size to explore associations between the two. Finally, although our study did not demonstrate a causal relationship between ECMO time and survival in ill COVID-19 patients, the difference in ECMO time between the surviving and dying populations could still suggest relevant studies for the following investigations.

Several limitations in our meta-analysis should be noted. First, relatively high heterogeneity was observed in our results. This may be due to the fact that our analysis was based on cohort studies. We included studies with both univariate and multivariate analyses, whose unadjusted confounders may have influenced our results. Second, despite the inclusion of a large number of studies, only a few articles reported effect sizes for the relationship between pre-ECMO or ECMO time and COVID-19 survival (*n* = 15). And the time periods varied across the studies we included, resulting in a small number of studies being included in each period (time from intubation or MV to ECMO = 3 for categorical variables and 7 for continuous variables). This may be another cause of bias and inaccurate results. In addition, the studies included in this analysis reported different effect sizes (OR, RR, and HR), making it impossible to report the pooled results due to differences in statistical methodology. It is important to continue to pay attention to relevant research and refine each analysis as needed in the future. Third, out of the 54 studies included, the majority were conducted in America. However, due to the limited number of studies included, subgroup analysis could not be conducted. Hence, the potential influence of confounding and potential intermediate factors, such as regional differences, study design, follow-up, and other clinical characteristics across studies, needs further investigation. Fourth, there were differences in the indicators for ECMO initiation in each study, with some studies following ELSO guidelines and others relying on decisions made by local experts, which may have impacted the results. Fifth, the varying definitions of death in each study and the inconsistent timing of these definitions, coupled with the short-term follow-up periods in most studies, may have led to an underestimation of reported mortality. Sixth, due to the observational nature of the analyses we included and the limited number of articles included, trial sequential analysis was not performed to assess the robustness of the findings and the need for further research. Finally, the meta-analysis is based on observational studies, so causality cannot be deduced from our study.

## 5. Conclusions

Based on current evidence, our results suggest that there are differences in pre-ECMO between COVID-19 survivors and non-survivors. We did not have sufficient evidence of a significant association between pre-ECMO time and survival in COVID-19 patients. In addition, non-survivors had a longer ECMO duration than survivors. Pre-ECMO time does not affect the timing of ECMO. Considering the limited evidence and possible bias, further studies in pre-ECMO and ECMO time on the survival of COVID-19 patients are needed to explore the association between them. Future guidelines may emphasize ECMO timing-specific risk assessment and management for severe COVID-19.

## Figures and Tables

**Table 1:** Basic characteristics of the articles included in the meta-analysis.

Author, Year, Country	Study Design	Data Source	Follow-Up Time, Sample Size	Mean Age (Years), Male (%)	ECMO Type	ECMO Initiation	Pre-ECMO Baseline Comorbidities	Other Treatment	Time Period	Survivor and Non-Survivor Time (Mean ± Standard Deviation; Day)	Estimate Effect (95% CI) and Adjusted
Akkanti, 2022, USA [19]	Retrospective cohort	Baylor St. Luke’s Medical Center, Houston Methodist Hospital, or University of Texas Memorial Herrmann Hospital between 15 March 2020, and 30 May 2020	2 years, 35	49, 43	VV ECMO and VA ECMO.	ELSO guidelines	66% obesity; 48% hypertension; 29% diabetes; 8% dialysis for AKI; 6% asthma	83% convalescent plasma; 31% remdesivir; 68% anti-IL-6 therapies	from intubation to ECMO	4.66 ± 3.52; 3.20 ± 2.38	
									ECMO duration	13.95 ± 11.74; 16.6 ± 15.77	
Alnababteh, 2021, USA [20]	Respective cohort	MedStar Washington Hospital Center from 23 March 2020 to 29 April 2020	Until ICU discharge, 13	45, 62	VV ECMO	EOLIA trial	38.5% hypertension; 30.8% diabetes	100% prone; 76.9% azithromycin; 76.9% hydro chloroquine; 69.2% IL-6 inhibitor; 100% neuromuscular blockade; 76.9% inhaled epoprostenol; 92.3% anticoagulation	From admission to ECMO	7.49 ± 4.02; 2.92 ± 4.34	
									ECMO duration	12.26 ± 4.29; 15.85 ± 11.61	
Bergman, 2021, USA [21]	Retrospective cohort	one of four adult ELSO-certified Centers of Excellence in Minnesota (University of Minnesota MHealth Fairview, Hennepin County Medical Center, Abbott Northwestern Hospital, and Mayo Clinic Rochester) from 1 March 2020 through 1 November 2020	60 days, 46	51, 83	VV ECMO and VAV ECMO	(1) On FiO_2_ ≥ 80%, PEEP ≥ 10, and VT ≤ 6 mL/kg PBW; (2) PaO_2_/FiO_2_ < 50 for more than 3 h; (3) PaO_2_/FiO_2_ < 80 for more than 6 h; (4) pH < 7.25, PaCO_2_ > 60 mmHg with RR > 35 for more than 6 h	41.3% obesity; 45.7% hypertension; 28.3% hyperlipidemia; 39.1% diabetes; 8.7% asthma/COPD; 6.5% CAD; 13% CKD	19.6% hydroxychloroquine + azithromycin; 67.4% remdesivir; 56.5% IL-6 inhibitor; 47.8% convalescent plasma; 45.7% steroids	ECMO duration	21.50 ± 14.79; 31.55 ± 17.88	
Beyls,2021, France [22]	Prospective cohort	Amiens University Hospital between 28 February 2020 and 1 June 2020, 1 September 2020 and 15 April 2020 and between 12 December 2020 and 15 June 2021	90 days, 54	58, 74	VV ECMO	ELSO guidelines	1% hypertension; 4% diabetes; 17% dyslipidemia; 7% chronic renal disease; 9% COPD/asthma; 7% coronary disease; 6% immunocompromised	81% glucocorticoids; 17% lopinavir-ritonavir; 7% hydroxychloroquine; 4% tocilizumab	From symptom onset to ECMO		HR 1.10 (1.02–1.17); multivariate adjustment
Biancari, 2021, Finland [23]	Retrospective cohort	ten ECMO centers in five European countries (France, Germany, Italy, Sweden, UK) between 2 March 2020 and 30 April 2020	6 months, 132	51, 83	VV ECMO, VA ECMO, VA ECMO and VVA ECMO	NA	11.4% dialysis; 22% diabetes; 3% cancer; 28.8% hypertension; 9.2% asthma/COPD; 0.8% congestive heart failure; 3% CAD; 1.5% stroke/transient ischemic attack	93.2% prone positioning; 4.5% convalescent plasma; 25% hydroxychloroquine/choloroquine; 6.8% extracorporeal cytokine absorber; 6.8% tocilizumab; 35.6% corticosteroids	from ICU admission to ECMO	5.30 ± 5.50; 6.20 ± 5.20	
									ECMO duration	15.10 ± 9.80; 14.10 ± 12.00	
Blazoski, 2021, USA [24]	Retrospective cohort	Thomas Jefferson University from 1 April 2020 to 11 June 2020	NA, 20	54, 60	VV ECMO and VA ECMO	NA	10% chronic lung disease; 30% diabetes; 5% liver failure; 5% chronic immunosuppression; 30% acute renal injury	40% steroids; 55% IL inhibitor; 20% remdesivir; 15% plasma	From symptom onset to ECMO	9.90 ± 4.60; 13.50 ± 8.40	
									From hospital admission to ECMO	12.00 ± 16.00; 8.80 ± 7.70	
									from MV to ECMO	8.58 ± 14.33; 3.17 ± 2.83	
									ECMO duration	11.00 ± 6.20; 17.00 ± 12.00	
Braaten, 2022, USA [25]	Prospective cohort	one of the four adult ECMO centers in the state of Minnesota (University of Minnesota, Hennepin County Medical Center, Abbott Northwestern Hospital, and the Mayo Clinic Rochester) from March 2020 to May 2021	60 days, 100	52, 75	VV ECMO	NA	32% hypertension; 28% hyperlipidemia; 32% diabetes; 11% asthma/COPD; 7% chronic artery disease; 11% CKD	23% hydroxychloroquine + azithromycin; 59% remdesivir; 39% IL-6 inhibitor; 46% convalescent plasma; 86% steroids	From hospital admission to ECMO	6.65 ± 3.81; 10.00 ± 6.11	
Casabella, 2021, Argentina [26]	Retrospective cohort	During the first surge in Argentina	NA, 26	50, 80	NA	NA	NA	NA	From symptom onset to ECMO	12.12 ± 5.25; 18.99 ± 11.72	
									From MV to ECMO	5.19 ± 4.81; 6.72 ± 5.66	
Charlon, 2021, UK [27]	Retrospective cohort	one of the commissioned UK respiratory ECMO centers from 1 April to 31 May 2020	NA, 34	44, 79	NA	NA	23.5% hypertension; 11.8% diabetes; 67.6% obese	NA	From MV to ECMO	5.20 ± 1.80; 4.60 ± 1.70	
									ECMO duration	11.10 ± 4.90; 15.60 ± 5.60	
Cheng, 2021, China [28]	Prospective cohort	62 authorized hospitals in Wuhan from 1 January 2020, to 1 May 2020	90 days, 74	57, 62	NA	(1) PaO_2_/FiO_2_ < 50 mmHg for more than 3 h; (2) PaO_2_/FiO_2_ < 80 mmHg for more than 6 h; (3) FiO_2_ = 1.0, PaO_2_/FiO_2_ < 100 mmHg; (4) pH < 7.25 and PaCO_2_ > 60 mmHg for more than 6 h, with RR > 35/min; (5) pH < 7.2 and plateau pressure > 30 cmH_2_O, even respiratory rate > 35/min; (6) severe air leakage syndrome	40.5% hypertension; 29.7% diabetes; 28.4% cardiovascular disease; 2.7% chronic pulmonary disease; 10.8% CKD; 10.8% chronic liver disease; 1.4% digestive disease; 9.5% cerebral vascular disease	39.2% prone position; 97.3% vasoactive drugs; 45.9% anti-viral drugs; 91.9% cortical steroids; 9.5% tocilizumab	From intubation to ECMO	3.80 ± 5.57; 5.53 ± 5.72	
									ECMO duration	10.54 ± 7.39; 15.35 ± 9.91	
Daviet, 2021, France [29]	Ambispective cohort	tertiary University Hospital of Assistance Publique-Hôpitaux de Marseille France, constituted of 5 different ICU units from 1 March 2020 to 31 May 2020 and 1 September 2020 to 30 November 2020	90 days, 76	60, 78	VV ECMO and VA ECMO	NA	38% obesity; 42.1% hypertension; 36.8% diabetes; 11.8% CAD; 21.1% dyslipidemia; 6.6% immunosuppression; 11.8% chronic lung disease; 5.3% chronic kidney disease	67% dexamethasone; 32% hydroxychloroquine; 4% antiviral treatment; 8% anti-IL 6; 16% anti-IL 1; 18% junus kinase 1/2 inhibitor; 62% high-dose corticosteroid	From ICU admission to ECMO	7.94 ± 5.39; 11.00 ± 4.63	OR 1.18 (0.98–1.40); adjusted for age, BMI, PRESERVE score, Charlson score
									From MV to ECMO	5.71 ± 4.62; 7.00 ± 6.17	
									ECMO duration	15.42 ± 10.78; 27.00 ± 18.51	
Diaz, 2021, Chile [30]	Retrospective cohort	Any of the 13 ECMO centers in Chile from 3 March through 31 August 2020	Until 3 March 2021, 85	48, 84	VV ECMO (three patients unclear)	Based on the ELSO COVID-19 guidelines	30.6% hypertension; 21.2% diabetes; 42.4% obesity; 7.1% COPD/asthma	91.8% prone positioning; 94.1% neuromuscular blockade	From intubation to ECMO	4.85 ± 4.57; 4.00 ± 3.10	HR 0.96 (0.90–1.02); univariate adjustment
Dreier, 2021, Germany [31]	Retrospective cohort	ICUs of the University Hospital Regensburg between 25 March and 7 May 2020	6 months, 16	58, 81	VV ECMO	NA	12.5% chronic pulmonary disease; 37.5% arterial hypertension; 6.3% CKD; 18.8% diabetes	NA	From symptom onset to ECMO	16.20 ± 5.94; 27.39 ± 5.03	
Gannon, 2022, USA (Derivation cohort) [32]	Retrospective cohort	International ELSO Registry data from at the time of initial data pull in April 2021	NA, 4553	50, 73	VV ECMO	NA	30% diabetes; 35.8% hypertension; 3.6% cardiac disease; 4% respiratory disease; 2.9% renal insufficiency; 9.6% central nervous system dysfunction; 4% immunocompromised; 1.8% cancer; 12.8% pneumothorax; 3.3% cardiac arrest; 28.1% acute renal failure	74.5% neuromuscular blocking agent; 60.4% prone positioning; 31.1% inhaled pulmonary vasodilators	ECMO duration	17.94 ± 14.91; 19.96 ± 15.50	
Gannon, 2022, USA (Validation cohort) [32]		International ELSO Registry data between April 2021 and October 2021	NA, 2582	47, 68	VV ECMO		24.2% diabetes; 34.8% hypertension; 2.5% cardiac disease; 3.8% respiratory disease; 2.4% renal insufficiency; 10.2% central nervous system dysfunction; 2.9% immunocompromised; 1.0% cancer; 15.7% pneumothorax; 3.1% cardiac arrest; 23.7% acute renal failure	70.6% neuromuscular blocking agent; 56.3% prone positioning; 31.4% inhaled pulmonary vasodilators	ECMO duration	21.99 ± 20.11; 22.97 ± 18.33	
Hajage, 2022, France [33]	Prospective cohort	University Hospitals of Geneva between 25 February 2020, and 4 May 2020	90 days, 269	53, 77	NA	Patients in ICU and on IMV, with time spent in ICU < 14 days (before ECMO initiation) and time spent on IMV < 7 days, age < 70 years, SAPS II at ICU admission 90 or less, and PaO_2_/FiO_2_,80 mmHg or PaCO_2_ < 60 mmHg	31% hypertension; 24% diabetes	89% prone position; 97% neuromuscular blockade; 50% NO; 22% corticosteroids	From MV to ECMO	5.00 ± 2.99; 6.70 ± 3.00	
									ECMO duration	13.05 ± 9.73; 9.35 ± 6.75	
Hall, 2022, USA [34]	Prospective cohort	multi-institutional SCOPE Registry database supported with ECMO at 45 hospitals located in 21 US states between 17 March 2020 and 11 October 2021	19 months, 505	48, 69	VV ECMO and VA ECMO	NA	13% asthma; 2.16% cancer; 7.68% chronic renal failure; 37.7% diabetes; 9.54% heart disease; 46.1% hypertension; 64% obesity	73.1% antiviral medication; 49.5% convalescent plasma; 12.6% hydroxychloroquine; 35.3% IL-6 blocker; 34.9% prostaglandin; 85.2% steroids	From COVID-19 diagnosis to ECMO	10.44 ± 8.03; 14.30 ± 7.45	
									From intubation to ECMO	3.41 ± 3.55; 4.00 ± 4.47	OR 0.82(0.49–1.38); multivariate adjustment
									ECMO duration	16.68 ± 12.55; 20.18 ± 13.78	
Haroun, 2022, USA [35]	Retrospective cohort	Montefiore Medical Center and other invited centers between 1 March 2020, and 30 April 2021	Until the time of discharge and/or transfer or in-hospital mortality, 37	44, 68	VV ECMO, VA ECMO, and VAV ECMO	NA	32% hypertension; 30% diabetes; 5% CAD	62% prone positioning; 72% vasopressors; 38% inotropes	From intubation to ECMO	1.46 ± 3.32; 3.07 ± 3.94	
									ECMO duration	22.49 ± 24.92; 24.18 ± 24.82	
Hermann, 2022, Austria [36]	Retrospective cohort	Medical University of Vienna, from January 2020 until May 2021	NA, 101	56, 70	VV ECMO, VA ECMO, and VVA ECMO	the official Medical University of Vienna consensus recommendations	59% arterial hypertension; 13% CAD; 35% obesity; 25% diabetes; 19% underlying pulmonary disease; 3% immunosuppression; 6% CKD	100% prone positioning; 21% inhaled NO; 6% tracheostomy	From MV to ECMO	7.59 ± 7.60; 7.79 ± 6.45	HR 0.93 (0.88–0.98); adjusted for the baseline condition of patients
									ECMO duration	18.55 ± 13.06; 15.21 ± 13.67	
Herrmann, 2022, Germany [37]	Retrospective cohort	26 ECMO centers across Germany between 1 January 2020 and 22 March 2021	NA, 673	NA, 79	VV ECMO, VA ECMO, and VVA ECMO	at the discretion of the respective centers according to their in-house standards	61.8% cardiovascular disease; 27.6% diabetes; 15.3% chronic pulmonary disease; 7.3% kidney disease	49.5% prone positioning; 92.1% therapeutic anticoagulation	From MV to ECMO	NA	OR 1.30 (0.76–2.22); adjusted for demographics, risk factors and comorbidities (age, sex, BMI, and immunosuppression within 6 months prior to admission), severity of disease (intubation prior to ECMO and EOLIA criteria), ECMO case volume, and complications (major bleeding or thromboembolic events, secondary bacterial infection, and renal replacement therapy)
									ECMO duration	14.98 ± 11.90; 12.83 ± 11.85	
Jacobs, 2022, USA [38]	Prospective cohort	29 hospitals in 18 states in the US from 17 March 2020 to 1 December 2020	NA, 200	50, 69	VV ECMO and VA ECMO	determined by the individual patient care teams at each of the contributing 29 hospitals	16.5% asthma; 3% cancer; 6% chronic renal failure; 38% diabetes; 11% heart disease; 47% hypertension; 64% obesity	63.3% prone position before ECMO; 35% tracheostomy; 54.5% antiviral medication; 52.4% convalescent plasma; 23% hydroxychloroquine; 38.4% IL-6 blocker; 41.7% prostaglandin; 72% steroids	From COVID-19 diagnosis to ECMO	9.10 ± 6.76; 12.80 ± 8.96	
									From intubation to ECMO	4.18 ± 3.80; 5.30 ± 5.30	
									ECMO duration	19.30 ± 16.40; 21.00 ± 15.90	
Kunavarapu, 2021, USA [39]	Retrospective cohort	a tertiary referral high-volume ECMO center between 3 October 2020 and 8 April 2020	NA, 52	48, 67	VV ECMO and VA ECMO	EOLIA trial	46.2% hypertension; 30.8% diabetes; 13.5% asthma	19.2% prior use of ACEi/ARB; 38.5% tracheostomy; 51.9% vasopressors; 76.9% convalescent plasma; 11.5% continuous renal replacement therapy	From symptom onset to ECMO	11.40 ± 4.80; 14.40 ± 5.50	
									From hospital admission to ECMO	4.70 ± 3.80; 6.40 ± 4.60	
									From intubation to ECMO	2.10 ± 2.60; 2.80 ± 2.60	OR 1.31 (1.0–1.7); multivariate adjustment
									ECMO duration	20.40 ± 24.50; 21.50 ± 17.90	
Lai, 2021, China [40]	Retrospective cohort	all adult COVID-19 patients (age from 35 to 91) from Beijing, Sichuan, Guangxi, Hunan, and Hebei province in China who received ECMO support between 3 February 2020, and 23 January 2021	Until died within 48 h or discharge, 50	66, 68	VV ECMO and VA ECMO	(1) developed a refractory severe ARDS; (2) Lung Injury Murray Score ≥ 3; (3) developed uncompensated hypercapnia with pH < 7.25 or PaCO_2_ > 60 mmHg over 6 h; (4) PaO_2_/FiO_2_ < 80 over 6 h; (5) PaO_2_/FiO_2_ < 50 mm Hg over 3 h	30% diabetes; 44% hypertension	NA	From symptom onset to ECMO	14.60 ± 10.73; 17.80 ± 15.98	
									From MV to ECMO	3.23 ± 3.18; 6.24 ± 7.41	
Lang, 2021, Germany [41]	Retrospective cohort	university hospital of Freiburg from 3 August 2020 to 4 August 2020	Until 28 May 2020, 34	67, 82	VV ECMO	evaluated by an interdisciplinary team of at least one ECMO specialist, a registered nurse, and a perfusionist following local standards	52.9% hypertension; 35.3% diabetes; 23.5% CAD; 17.6% other cardiac disease; 23.5% CKD; 23.5% cancer; 11.8% immunosuppression	88.2% positioning maneuvers; 35.3% renal replacement therapy; 82.4% vasopressor therapy	ECMO duration	16.00 ± 7.00; 22.00 ± 30.00	
Lebreton, 2021, France [42]	Retrospective cohort	any Greater Paris ICU between 8 March and 3 June 2020	90 days, 302	52, 78	VV ECMO, VA ECMO and VAV ECMO	EOLIA trial	34% hypertension; 29% diabetes; 3% ischemic cardiomyopathy; 11% chronic respiratory disease; 6% immunocompromised	96% neuromuscular blockade; 94% prone positioning; 56% inhaled NO or prostacyclin; 20% steroids; 12% renal replacement therapy; 20% tracheostomy	From symptom onset to ECMO	13.35 ± 5.24; 14.70 ± 5.98	
									From hospital admission to ECMO	6.35 ± 3.75; 7.70 ± 4.49	
									From intubation to ECMO	4.00 ± 3.00; 5.35 ± 3.74	OR 0.91 (0.84–0.99); multivariate adjustment
									ECMO duration	18.05 ± 12.74; 14.46 ± 14.21	
Lee, 2022, Republic of Korea [43]	Retrospective cohort	1200-bed tertiary academic hospital and ECMO referral center in Republic of Korea from January 2020 to December 2021	NA, 39	64, 59	VV ECMO, VA ECMO and VAV ECMO	Decided by consulting with the internal medicine department	51.3% hypertension; 41% diabetes; 2.6% COPD; 10.3% heart failure; 2.6% liver cirrhosis; 2.6% CKD; 10.3% malignancy	61.5% remdesivir; 79.5% antibiotics; 74.4% vasopressor; 97.4% steroid. 17.9% tocilizumab; 25.6% continuous renal replacement therapy	From MV to ECMO	1.10 ± 2.49; 12.61 ± 6.67	
									ECMO duration	12.64 ± 7.06; 19.43 ± 21.02	
Levy, 2022, France [44]	Retrospective cohort	A 50-bed mixed ICU from October 2020 to June 2021	7 days, 11	52, 82	VV ECMO	NA	27.3% diabetes; 18.2% COPD; 18.2% coronary disease	NA	From symptom onset to ECMO	21.78 ± 23.13; 18.57 ± 10.01	
									From ICU admission to ECMO	16.11 ± 22.12; 11.38 ± 10.49	
									From MV to ECMO	8.42 ± 19.61; 8.35 ± 9.53	
									ECMO duration	21.89 ± 22.12; 26.24 ± 13.35	
Loforte, 2021, Italy [45]	Retrospective cohort	12 ECMO hub venters across Italy between 1 March and 15 September 2020	Until 30 September 2020, 71	55, 86	VV ECMO and VAV ECMO	a multidisciplinary team assessment	16.9% diabetes; 43.7% hypertension; 8.5% CAD; 7% atrial fibrillation; 2.8% concomitant heart disease; 7% asthma/COPD; 4.2% CKD; 2.8% dialysis	15.5% ACEi; 8.5% ARBs; 100% IMV; 70.4% antiretroviral therapy; 85% prone positioning; 85% neuromuscular blockade; 19.7% epinephrine; 77.5% norepinephrine; 18.3% inhaled pulmonary vasodilators	From MV to ECMO	4.99 ± 4.00; 4.23 ± 3.68	
									ECMO duration	14.36 ± 14.90; 15.71 ± 9.19	
Maharaj, 2022, USA [46]	Retrospective cohort	University of Minnesota between January 2020 and December 2020	NA, 17	48, 65	VV ECMO	NA	65% diabete; 53% hypertension; 12% COPD	35% home ACEi/ARB/ARNI use; 76% prone positioning	From hospital admission to ECMO	5.44 ± 5.42; 9.78 ± 5.76	
									ECMO duration	18.76 ± 12.86; 30.85 ± 18.06	
Mongero, 2021, USA [47]	Retrospective cohort	40 institutions from SCOPE between 18 March 2021	Until discharge, 342	49, 71	VV ECMO and VA ECMO	NA	14.9% asthma; 2.68% cancer; 8.46% chronic renal failure; 37.6% diabetes; 10.7% heart disease; 47.9% hypertension	67.5% prone before ECMO	From COVID-19 diagnosis to ECMO	11.25 ± 9.03; 13.70 ± 9.72	
									From intubation to ECMO	4.15 ± 3.88; 4.86 ± 4.73	
									ECMO duration	19.80 ± 15.50; 23.40 ± 19.10	
Natanov, 2022, Germany [48]	Retrospective cohort	Hannover medical school center between January 2020 and August 2021	NA, 85	55, 84	VV ECMO	ELSO guidelines	16.5% COPD; 23.5% II diabetes mellitus; 15.3% cardiovascular disease; 10.6% renal insufficiency; 47.1% arterial hypertension; 81.2% obesity	96.5% administration of antibiotics; 23.5% administration of antivirals; 1.2% inotropes; 74.1% vasopressors; 87.1% prone position	From hospital admission to ECMO	8.10 ± 1.30; 12.10 ± 1.20	
									ECMO duration	12.40 ± 1.50; 18.20 ± 1.90	
Nesserler, 2022, France [49]	Prospective cohort	ECMOSARS registry from before 21 April 2020 up to 25 October 2020	90 days, 429	53, 79	VA ECMO and VV ECMO	NA	38% hypertension; 30% diabetes; 3% COPD; 3% chronic respiratory failure; 1% congestive heart failure; 5% CAD; 4% CKD; 2% cancer	6% steroids; 2% NSAIDs; 10% ACEi; 14% ARBs	From MV to ECMO		HR 1.74 (1.07–2.83); adjusted for patient-related confounders (sex, age, BMI, diabetes, COPD, chronic respiratory failure, congestive heart failure, CKD, malignancy, and previous corticotherapy) and pre-ECMO hospitalization-related confounders (septic shock, total bilirubin at cannulation, pH at cannulation, PaCO_2_ fractional inspired oxygen tension (FiO_2_ at cannulation, PaO_2_) ratio at cannulation, driving pressure, left ventricular ejection fraction, ventilator-associated pneumonia, and delay from hospitalization to ICU admission)
									ECMO duration	14.05 ± 9.70; 12.75 ± 11.19	
Olivier, 2021, France [50]	Retrospective cohort	three French ECMO centers from March 2020 to June 2021	NA, 56	58, 88	VV ECMO	EOLIA trial criteria	52% hypertension; 29% diabetes	100%prone position; l00% neuromuscular blockers	From symptom onset to ECMO	15.86 ± 7.83; 18.71 ± 9.36	
									From intubation to ECMO	6.71 ± 9.39; 6.71 ± 4.68	
									ECMO duration	19.07 ± 18.00; 20.57 ± 15.59	
Pacheco, 2020, Spain [51]	Prospective cohort	Vall d’Hebron University Hospital from 15 March to 30 July 2020	NA, 24	52, 58	Mainly VV ECMO	PaO_2_/FiO_2_ < 80 mmHg, refractory to prone position, and/or PaCO_2_ > 80 mmHg and pH < 7.25 for >6 h	NA	NA	From MV to ECMO		OR 1.31 (1.11–1.67); univariate adjustment
Powell, 2022, USA [52]	Retrospective cohort	Shock Trauma Center and the University of Maryland Medical Center from 1 January 2020, to 28 July 2021	Until discharge, 93	44, 71	VV ECMO	NA	10.8% asthma/COPD; 23.7% diabetes; 1.1% congestive heart failure; 1.1% liver disease	70.9% vasopressor; 18.3% inotrope; 67.7% prone position; 95.7% paralysis; 16.1% inhaled pulmonary vasodilator; 78.5% steroids; 39.8% convalescent plasma; 60.2% remdesivir; 23.7% monoclonal antibody	From COVID-19 diagnosis to ECMO	8.56 ± 7.59; 11.00 ± 4.67	
									From intubation to ECMO	2.71 ± 3.03; 2.36 ± 2.34	
Rabie, 2021, Saudi Arabia [53]	Retrospective cohort	19 ECMO centers in five countries of the SWAAC-ELSO region between 1 March 2020, and 30 September 2020	Discharge or decannulation, 307	45, 81	VA ECMO, VV ECMO, and VAV ECMO	ELSO guidelines	31.9% diabetes; 15.3% hypertension; 5.9% COPD/asthma; 2.6% ischemic heart disease	58.3% vasopressor; 52.1% prone	Pre-ECMO MV		OR 1.68 (0.90–3.19); multivariate adjustment
Raff, 2020, USA [54]	Retrospective cohort	University of North Carolina Medical Center from 1 April to 31 July 2020	NA, 25	47, 72	VV ECMO	NA	48% diabetes	NA	From symptom onset to ECMO	12.40 ± 8.40; 19.90 ± 7.30	
									From ICU admission to ECMO		RR 1.04 (1.01–1.09); multivariate adjustment
									From MV to ECMO	3.50 ± 5.50; 6.10 ± 4.20	
Rajajee, 2020, USA [55]	Prospective cohort	University of Michigan Medical School from 1 March 2020, to 31 July 2020	1 year, 23	45, 65	VV ECMO and VA ECMO	persistent severe hypoxemia despite maximal MV and rescue approaches and no absolute contraindications presents	NA	70% continuous renal replacement therapy; 39% hemodialysis; 35% glucocorticoids; 22% tocilizumab; 9% remdesivir	From symptom onset to ECMO	14.73 ± 9.88; 18.00 ± 10.50	
									From intubation to ECMO	8.09 ± 4.11; 6.00 ± 7.00	
									ECMO duration	16.00 ± 6.59; 19.88 ± 38.05	
Riera, 2022, Spain [56]	Retrospective-prospective cohort study	24 ECMO centers (22 in Spain and two in Portugal) from 1 March to 1 December 2020	6 months, 319	53, 81	NA	EOLIA trial	37.9% hypertension	NA	From symptom onset to ECMO		HR 1.009 (0.991–1.027); univariate adjustment
									From MV to ECMO		HR 1.028 (1.003–1.053); multivariate adjustment
Saeed, 2022, USA [57]	Retrospective cohort	Montefiore Medical Center invited 17 centers between 1 March 2020 and 30 April 2021	90 days, 435	48, 71	VV ECMO	NA	65% hypertension; 50% diabetes; 4% chronic respiratory disease; 2% malignant neoplasm; 6% CAD;	77% prone positioning; 61% vasopressors	From intubation to ECMO		HR 1.01 (0.98–1.03); adjusted for age, sex, BMI, cardiopulmonary resuscitation prior to ECMO, transferred to ECMO hospital, prone position prior to ECMO, time from symptoms to intubation, and PaCO_2_ before ECMO placement, use of intravenous steroids
Saeed, 2022, USA [58]	Retrospective cohort	Montefiore Medical Center invited 17 centers between 1 March 2020 and 30 September 2020	90 days, 292	49, 72	VV ECMO, VA ECMO and VAV ECMO	NA	41% hypertension; 31% diabetes; 3% chronic respiratory disease; 1% malignant neoplasm; 4% CAD;	77% prone positioning; 64% vasopressors	From intubation to ECMO	3.00 ± 3.00; 3.65 ± 3.75	
									ECMO duration	16.41 ± 11.99; 21.81 ± 21.04	
Schmidt, 2020, France [59]	Retrospective cohort	Paris–Sorbonne University Hospital Network ICUs (three at La Pitié–Salpêtrière Hospital, one in Saint-Antoine Hospital, and one in Tenon Hospital) from 8 March to 2 May 2020	60 days, 83	49, 73	VV ECMO, VA ECMO and VAV ECMO	(1) partial pressure of arterial oxygen over a FiO_2_ ratio of less than 50 mmHg for more than 3 h; (2) PaO_2_/FiO_2_ less than 80 mmHg for more than 6 h; or (3) arterial blood pH less than 7.25 with a partial pressure of arterial CO_2_ of 60 mmHg or more for 6 h or more	39% hypertension; 31% diabetes; 5% ischemic cardiomyopathy; 11% chronic respiratory disease/COPD/asthma; 4% immunocompromised	96% neuromuscular blockade; 94% prone positioning; 34% inhaled NO or prostacyclin; 7% steroids; 1% almitrine; 5% renal replacement therapy	From intubation to ECMO	3.65 ± 2.29; 6.00 ± 3.11	
Schmidt, 2021, France [60]	Retrospective cohort	Paris–Sorbonne University Hospital Network ICUs from 8 March 2020, to 28 January 2021	90 days, 159	51, 72	VV ECMO and VA ECMO	EOLIA trial respiratory severity criteria	40% hypertension; 34% diabetes; 15% chronic respiratory disease; 6% immunocompromise	94% neuromuscular blockade; 92% prone positioning; 43% inhaled NO/prostacyclin; 6% high-dose corticosteroids; 0.6% almitrine; 3% renal replacement therapy	From ICU admission to ECMO		HR 3.02 (1.15–7.92); multivariate adjustment
Shaefi, 2021, USA [61]	Retrospective cohort	data from the STOPCOVID (ICUs at 55 geographically diverse hospitals across the US) between 1 March and 1 July 2020	Until discharge, death or 1 September 2020 (minimum of 60 days), 190	49, 72	VV ECMO	PaO_2_/FiO_2_ ratio < 100 mmHg while receiving IMV	6.8% chronic lung disease; 3.7% chronic artery disease; 2.1% chronic liver disease; 1.1% end-stage renal disease; 1.6% active malignancy; 62.6% any chronic condition	78.4% IMV; 71.1% prone positioning; 78.4% neuromuscular blockade; 15.8% inhaled NO; 19% inhaled epoprostenol; 71.6% anticoagulation	From symptom onset to ECMO	12.70 ± 4.50; 13.35 ± 6.83	
									From hospital admission to ECMO	5.00 ± 3.00; 7.06 ± 5.31	
									From ICU admission to ECMO	2.65 ± 3.75; 3.65 ± 3.79	
									From MV to ECMO	2.35 ± 3.75; 3.35 ± 3.79	
									ECMO duration	16.70 ± 10.50; 15.65 ± 9.86	
Supady, 2021, Germany [62]	Retrospective cohort	15 centers in the US, Germany, Belgium, Switzerland, and Italy from 12 March 2020, through 5, June 2020	30 days, 127	59, 79	VV ECMO	NA	2% heart failure NYHA IV; 10% chronic lung disease; 9% dialysis-dependent kidney failure; 5% hematologic malignancy; 2% solid malignant tumor; 6% immunosuppressive therapy	74% prone positioning before ECMO; 5% NO use; 9% bicarbonate use; 53% neuromuscular blockers; 19% renal replacement therapy	From MV to ECMO	4.06 ± 5.30; 7.06 ± 5.70	
Takeuchi, 2022, Japan [63]	Retrospective cohort	Osaka Prefecture between 29 January and 9 November 2020	Ended on the day of ECMO termination of died, 39	NA, 92	NA	NA	41% comorbidities	NA	From symptom onset to ECMO	14.00 ± 6.29; 11.64 ± 4.12	
									ECMO duration	14.64 ± 7.08; 9.64 ± 9.06	
Trejnowska, 2022, Poland [64]	Retrospective cohort	four Polish ECMO centers between 1 March 2020, and 31 May 2021	NA, 158	46, 75	Mainly VV ECMO	persistent hypoxemia with PaO_2_/FiO_2_ < 150 mmHg and/or respiratory acidosis with pH < 7.25 and PaCO_2_ > 60 mmHg	25.9% arterial hypertension; 7% chronic pulmonary disease; 2.5% cancer; 4.4% psychiatric disorders; 3.8% thyroid dysfunction; 12.7% diabetes; 1.9% chronic heart failure; 3.8% CAD;	NA	From hospital admission to ECMO	6.30 ± 5.70; 8.10 ± 6.10	
									From MV to ECMO	4.80 ± 5.20; 5.60 ± 6.50	
Varghese, 2021, USA [65]	Retrospective cohort	MedStar Washington Hospital Center from April 2020 through December 2020	NA, 32	45, 69	VV ECMO	NA	62.5% hypertension; 37.5% diabetes	NA	From symptom onset to ECMO	18.00 ± 5.70; 12.00 ± 7.00	
Vigneshwar, 2022, USA [66]	Prospective cohort	4 ECMO referral centers between March and October 2020	NA, 51	50, 65	VV ECMO	International ELSO guidelines	5.88% COPD; 39.21% essential hypertension; 39.21% diabetes; 50.98% peripheral artery disease; 3.92% stroke/transient ischemic attack; 19.61% asthma; 7.84% central nervous system dysfunction	29.41% inotrope; 37.25% steroids; 21.57% cytokine blocker; 52.94% remdesivir; 35.29% hydrochloroquine/chloroquine; 60.78% convalescent plasma	From hospital admission to ECMO	5.57 ± 6.21; 3.92 ± 5.61	
									From MV to ECMO	5.86 ± 4.35; 3.78 ± 4.00	
									ECMO duration	12.42 ± 7.76; 18.08 ± 10.41	
Yang, 2020, China [67]	Retrospective cohort	Twenty-one ICUs in Hubei since 1 January	Up to 31 May 2020 73	60, 63	VV ECMO	NA	13.7% CAD; 37% hypertension; 17.8% diabetes; 6.8% COPD; 1.4% malignancy	23.7% renal replacement therapy; 66.1% prone position ventilation; 86.4% steroid therapy; 11.8% convalescent plasma	From symptom to ECMO	24.00 ± 8.24; 24.41 ± 10.64	
									From MV to ECMO	2.59 ± 4.94; 4.00 ± 4.56	
Yaqoob, 2022, USA [68]	Retrospective cohort	The ICUs of a quaternary care hospital between 3 January 2020 and 31 August 2021	NA, 31	47, 65	NA	NA	NA	NA	From MV to ECMO	2.92 ± 3.82; 4.86 ± 5.19	
Yoshino, 2021, Japan [69]	Retrospective cohort	Fukuoka University Hospital ECMO Center between April 2020 and December 2020	NA, 19	61, 84	NA	NA	NA	NA	ECMO duration	8.18 ± 5.45; 40.81 ± 56.95	
Zaaqoq, 2022, USA [70]	Retrospective cohort	quaternary care institution from 1 April 2020, to 1 January 2021	NA, 32	44, 69	VV ECMO	EOLIA trial criteria	46.9% obesity; 37.5% diabetes; 34.4% hypertension; 3.1% malignancy	31.25% hydroxychloroquine; 28.13% azithromycin; 40.6% IL-6 inhibitor; 15.6% intravenous steroids; 33.3% remdesivir; 21.9% convalescent plasma	From symptom onset to ECMO	15.55 ± 6.47; 9.73 ± 8.18	
									From MV to ECMO	2.64 ± 2.43; 2.45 ± 3.27	
									ECMO duration	12.00 ± 15.88; 22.09 ± 23.72	
Zayat, 2021, Germany [71]	Retrospective cohort	RWTH Aachen University Hospital from 1 March 2020, to 20 April 2020	NA, 17	57, 65	VV ECMO and VA ECMO	ELSO	35% hypertension; 6%CAD; 35% diabetes; 82% kidney disease; 6% peripheral arterial disease; 29% prior pneumonia; 18% COPD; 35% atrial fibrillation; 6% malignancy	18% antiviral treatment; 70.6% inotropes; 88.2% vasopressor; 47% inhaled NO inhalation	From hospital admission to ECMO	4.00 ± 1.75; 11.81 ± 12.06	
									From MV to ECMO	3.37 ± 0.87; 9.25 ± 13.40	
									ECMO duration	15.26 ± 7.00; 15.44 ± 11.17	
Zhang, 2020, UK [72]	Retrospective cohort	GSTFT in London between 3 March and 2 May 2020	NA, 43	45, 77	VV ECMO	NA	48.8% obesity; 23.3% hypertension; 18.6% diabetes; 11.6% asthma	NA	From MV to ECMO	4.29 ± 3.12; 3.64 ± 4.12	
									ECMO duration	14.36 ± 10.14; 12.05 ± 10.09	

Abbreviations: ECMO: extracorporeal membrane oxygenation; USA: the United States of America; VV: venovenous; VA: venoarterial; ELSO: Extracorporeal Life Support Organization; AKI: acute kidney injury; IL: interleukin; ICU: intensive care unit; EOLIA: ECMO to Rescue Lung Injury in Severe ARDS; VAV: venoarterio-venous; FiO_2_: fraction of inspiration O_2_; PEEP: positive end-expiratory pressure; VT: tidal volume; ml: milliliter; kg: kilogram; PBW: predicted body weight; PaO_2_: partial pressure of oxygen; mmHg: millimeter of mercury; pH: hydrogen ion concentration; PaCO_2_: partial pressure of carbon dioxide; RR: respiratory rate; COPD: chronic obstructive pulmonary disease; CAD: coronary artery disease; CKD: chronic kidney disease; HR: hazard ratio; UK: United Kingdom of Great Britain and Northern Ireland; VVA: veno-venoarterial; NA: not available; MV: mechanical ventilation; OR: odds ratio; BMI: body mass index; PRESERVE: PRedictiong dEath for SEvere ARDS on veno-venous ECMO; IMV: invasive mechanical ventilation; SAPS: simplified acute physiology score; NO: nitric oxide; SCOPE: SpecialtyCare Operative Procedural rEgistry; US: United States; ACEi: angiotensin-converting enzyme inhibitors; ARB: angiotensin II receptor blocker; ARDS: acute respiratory distress syndrome; ARNI: angiotensin receptor neprilysin inhibitor; COVID-19: coronavirus disease 2019; ECMOSARS: The Extracorporeal Membrane Oxygenation for Respiratory Failure and/or Heart failure related to Severe Acute Respiratory Syndrome Coronavirus 2; NSAID: nonsteroidal anti-inflammatory drug; SWAAC-ELSO: The South Asia, West Asia, and Africa Chapter of Extracorporeal Life Support Organization; RR: relative risk; STOPCOVID: Study of the Treatment and Outcomes in Critically Ill Patients with COVID-19; NYHA II: New York Heart Association II; GSTFT: Guy’s and St Thomas’ NHS Foundation Trust.

## Data Availability

The datasets used and analyzed during the current study are available from the corresponding author on reasonable request.

## Supplementary Materials

The following supporting information can be downloaded at: https://www.mdpi.com/article/10.3390/jcm13030868/s1, Table S1: guidelines of the Preferred Reporting Item for Systematic Review and Meta-Analysis 2020 (PRISMA 2020); Table S2: Detailed description of the search strategy; Table S3: Studies excluded (n = 150) with reasons; Table S4. Quality assessment of included studies; Figure S1: (A). Forest plot showing the differences in time from COVID-19 diagnosis to ECMO between survivors and non-survivors in COVID-19 patients; (B). Forest plot showing the differences in time from hospital admission to ECMO between survivors and non-survivors in COVID-19 patients; (C). Forest plot showing the differences in time from ICU admission to ECMO between survivors and non-survivors in COVID-19 patients; D. Forest plot for the association between time from intubation or MV to ECMO and survival in COVID-19 patients. (a). Forest plot showing the differences in time between survivors and non-survivors in COVID-19 patients. (b). Forest plot for the association between time and survival, analyzed as category variables (highest vs. lowest). (c). Forest plot for the association between time and survival, analyzed as continuous variables (per one-day increase); Figure S2. Regression analysis of pre-ECMO time and ECMO duration. (a). Survivors; (b). Non-survivors; Figure S3. Sensitivity analysis of time from MV or intubation to ECMO difference in COVID-19 for mortality by omitting one study at once. (a). Category variables; (b). Continuous OR variables; (c). Continuous HR variables.
