# Supplementary material for: Relationship between the Pre-ECMO and ECMO Time and Survival of Severe COVID-19 Patients: A Systematic Review and Meta-Analysis
Source: J Clin Med. 2024 Feb 1;13(3):868. doi: 10.3390/jcm13030868 (PMC10856383; doi:10.3390/jcm13030868)
Supplement: Supplementary file 1 [file jcm-13-00868-s001.zip › jcm-2770704-supplementary.pdf]

Supplemental Material

Table S1. guidelines of the Preferred Reporting Item for Systematic Review and Meta-Analysis 2020 (PRISMA 2020).

Section and Topic	Item #	Checklist item	Location where item is reported
**TITLE**			
Title	1	Identify the report as a systematic review.	1
**ABSTRACT**			
Abstract	2	See the PRISMA 2020 for Abstracts checklist.	5-33
**INTRODUCTION**			
Rationale	3	Describe the rationale for the review in the context of existing knowledge.	36-62
Objectives	4	Provide an explicit statement of the objective(s) or question(s) the review addresses.	63-68
**METHODS**			
Eligibility criteria	5	Specify the inclusion and exclusion criteria for the review and how studies were grouped for the syntheses.	93-101
Information sources	6	Specify all databases, registers, websites, organisations, reference lists and other sources searched or consulted to identify studies. Specify the date when each source was last searched or consulted.	77-82
Search strategy	7	Present the full search strategies for all databases, registers and websites, including any filters and limits used.	83-87
Selection process	8	Specify the methods used to decide whether a study met the inclusion criteria of the review, including how many reviewers screened each record and each report retrieved, whether they worked independently, and if applicable, details of automation tools used in the process.	89-105
Data collection process	9	Specify the methods used to collect data from reports, including how many reviewers collected data from each report, whether they worked independently, any processes for obtaining or confirming data from study investigators, and if applicable, details of automation tools used in the process.	107-111
Data items	10a	List and define all outcomes for which data were sought. Specify whether all results that were compatible with each outcome domain in each study were sought (e.g. for all measures, time points, analyses), and if not, the methods used to decide which results to collect.	96-97
	10b	List and define all other variables for which data were sought (e.g. participant and intervention characteristics, funding sources). Describe any assumptions made about any missing or unclear information.	96-97
Study risk of bias assessment	11	Specify the methods used to assess risk of bias in the included studies, including details of the tool(s) used, how many reviewers assessed each study and whether they worked independently, and if applicable, details of automation tools used in the process.	112-115
Effect measures	12	Specify for each outcome the effect measure(s) (e.g. risk ratio, mean difference) used in the synthesis or presentation of results.	122-130
Synthesis methods	13a	Describe the processes used to decide which studies were eligible for each synthesis (e.g. tabulating the study intervention characteristics and comparing against the planned groups for each synthesis (item #5)).	93-101
	13b	Describe any methods required to prepare the data for presentation or synthesis, such as handling of missing summary statistics, or data conversions.	117-121
	13c	Describe any methods used to tabulate or visually display results of individual studies and syntheses.	122-130
	13d	Describe any methods used to synthesize results and provide a rationale for the choice(s). If meta-analysis was performed, describe the model(s), method(s) to	122-130

Section and Topic	Item #	Checklist item	Location where item is reported
		identify the presence and extent of statistical heterogeneity, and software package(s) used.	
	13e	Describe any methods used to explore possible causes of heterogeneity among study results (e.g. subgroup analysis, meta-regression).	135-140
	13f	Describe any sensitivity analyses conducted to assess robustness of the synthesized results.	135-140
Reporting bias assessment	14	Describe any methods used to assess risk of bias due to missing results in a synthesis (arising from reporting biases).	135-140
Certainty assessment	15	Describe any methods used to assess certainty (or confidence) in the body of evidence for an outcome.	135-140
**RESULTS**			
Study selection	16a	Describe the results of the search and selection process, from the number of records identified in the search to the number of studies included in the review, ideally using a flow diagram.	144-155
	16b	Cite studies that might appear to meet the inclusion criteria, but which were excluded, and explain why they were excluded.	148-155
Study characteristics	17	Cite each included study and present its characteristics.	157-175
Risk of bias in studies	18	Present assessments of risk of bias for each included study.	177-181
Results of individual studies	19	For all outcomes, present, for each study: (a) summary statistics for each group (where appropriate) and (b) an effect estimate and its precision (e.g. confidence/credible interval), ideally using structured tables or plots.	184-234
Results of syntheses	20a	For each synthesis, briefly summarise the characteristics and risk of bias among contributing studies.	184-234
	20b	Present results of all statistical syntheses conducted. If meta-analysis was done, present for each the summary estimate and its precision (e.g. confidence/credible interval) and measures of statistical heterogeneity. If comparing groups, describe the direction of the effect.	184-234
	20c	Present results of all investigations of possible causes of heterogeneity among study results.	184-234
	20d	Present results of all sensitivity analyses conducted to assess the robustness of the synthesized results.	184-234
Reporting biases	21	Present assessments of risk of bias due to missing results (arising from reporting biases) for each synthesis assessed.	184-234
Certainty of evidence	22	Present assessments of certainty (or confidence) in the body of evidence for each outcome assessed.	236-241
**DISCUSSION**			
Discussion	23a	Provide a general interpretation of the results in the context of other evidence.	245-253
	23b	Discuss any limitations of the evidence included in the review.	353-377
	23c	Discuss any limitations of the review processes used.	353-377
	23d	Discuss implications of the results for practice, policy, and future research.	316-336
**OTHER INFORMATION**			
Registration and protocol	24a	Provide registration information for the review, including register name and registration number, or state that the review was not registered.	71-75
	24b	Indicate where the review protocol can be accessed, or state that a protocol was not prepared.	71-75
	24c	Describe and explain any amendments to information provided at registration or in the protocol.	71-75
Support	25	Describe sources of financial or non-financial support for the review, and the role of the funders or sponsors in the review.	

Section and Topic	Item #	Checklist item	Location where item is reported
Competing interests	26	Declare any competing interests of review authors.	
Availability of data, code and other materials	27	Report which of the following are publicly available and where they can be found: template data collection forms; data extracted from included studies; data used for all analyses; analytic code; any other materials used in the review.	

From: Page MJ, McKenzie JE, Bossuyt PM, Boutron I, Hoffmann TC, Mulrow CD, et al. The PRISMA 2020 statement: an updated guideline for reporting systematic reviews. BMJ 2021;372:n71. doi: 10.1136/bmj.n71. For more information, visit: http://www.prisma-statement.org/.

Table S2. Detailed description of the search strategy.

**PubMed**	
#1	COVID-19 [MeSH Terms]
#2	'COVID 19' OR 'SARS-CoV-2 Infection' OR 'SARS CoV 2 Infection' OR 'SARS-CoV-2 Infections' OR '2019 Novel Coronavirus Disease' OR '2019 Novel Coronavirus Infection' OR '2019-nCoV Disease' OR '2019 nCoV Disease' OR '2019-nCoV Diseases' OR 'COVID-19 Virus Infection' OR 'COVID 19 Virus Infection' OR 'COVID-19 Virus Infections' OR 'Coronavirus Disease 2019' OR 'Coronavirus Disease-19' OR 'Coronavirus Disease 19' OR 'Severe Acute Respiratory Syndrome Coronavirus 2 Infection' OR 'SARS Coronavirus 2 Infection' OR 'COVID-19 Virus Disease' OR 'COVID 19 Virus Disease' OR 'COVID-19 Virus Diseases' OR '2019-nCoV Infection' OR '2019 nCoV Infection' OR '2019-nCoV Infections' OR 'COVID19' OR 'COVID-19 Pandemic' OR 'COVID 19 Pandemic' OR 'COVID-19 Pandemics'
#3	Extracorporeal Membrane Oxygenations [MeSH Terms]
#4	'Extracorporeal Membrane Oxygenations' OR 'ECLS Treatment' OR 'ECLS Treatments' OR 'Extracorporeal Life Support' OR 'Extracorporeal Life Supports' OR 'ECMO Treatment' OR 'ECMO Treatments' OR 'ECMO Extracorporeal Membrane Oxygenation' OR 'Venoarterial ECMO' OR 'Venoarterial ECMOs' OR 'Venoarterial Extracorporeal Membrane Oxygenation' OR 'Venovenous ECMO' OR 'Venovenous ECMOs' OR 'Venovenous Extracorporeal Membrane Oxygenation'
#5	Outcome [MeSH Terms]
#6	'Prognos*' OR 'Surviv*' OR 'Mortalit*' OR 'Fatal*' OR 'Outcome'
#7	#1 OR #2
#8	#3 OR #4
#9	#5 OR #6
#10	#7 AND #8 AND #9
**Embase**	
#1	'COVID 19' OR 'SARS-CoV-2 Infection' OR 'SARS CoV 2 Infection' OR 'SARS-CoV-2 Infections' OR '2019 Novel Coronavirus Disease' OR '2019 Novel Coronavirus Infection' OR '2019-nCoV Disease' OR '2019 nCoV Disease' OR '2019-nCoV Diseases' OR 'COVID-19 Virus Infection' OR 'COVID 19 Virus Infection' OR 'COVID-19 Virus Infections' OR 'Coronavirus Disease 2019' OR 'Coronavirus Disease-19' OR 'Coronavirus Disease 19' OR 'Severe Acute Respiratory Syndrome Coronavirus 2 Infection' OR 'SARS Coronavirus 2 Infection' OR 'COVID-19 Virus Disease' OR 'COVID 19 Virus Disease' OR 'COVID-19 Virus Diseases' OR '2019-nCoV Infection' OR '2019 nCoV Infection' OR '2019-nCoV Infections' OR 'COVID19' OR 'COVID-19 Pandemic' OR 'COVID 19 Pandemic' OR 'COVID-19 Pandemics'
#2	'Extracorporeal Membrane Oxygenations' OR 'ECLS Treatment' OR 'ECLS Treatments' OR 'Extracorporeal Life Support' OR 'Extracorporeal Life Supports' OR 'ECMO Treatment' OR 'ECMO Treatments' OR 'ECMO Extracorporeal Membrane Oxygenation' OR 'Venoarterial ECMO' OR 'Venoarterial ECMOs' OR 'Venoarterial Extracorporeal Membrane Oxygenation' OR 'Venovenous ECMO' OR 'Venovenous ECMOs' OR 'Venovenous Extracorporeal Membrane Oxygenation'
#3	'Prognos*' OR 'Surviv*' OR 'Mortalit*' OR 'Fatal*' OR 'Outcome'
#4	#1 AND #2 AND #3
**Cochrane**	
#1	MeSH descriptor: [COVID-19] explode all trees
#2	'COVID 19' OR 'SARS-CoV-2 Infection' OR 'SARS CoV 2 Infection' OR 'SARS-CoV-2 Infections' OR '2019 Novel Coronavirus Disease' OR '2019 Novel Coronavirus Infection' OR '2019-nCoV Disease' OR '2019 nCoV Disease' OR '2019-nCoV Diseases' OR 'COVID-19 Virus Infection' OR 'COVID 19 Virus Infection' OR

	'COVID-19 Virus Infections' OR 'Coronavirus Disease 2019' OR 'Coronavirus Disease-19' OR 'Coronavirus Disease 19' OR 'Severe Acute Respiratory Syndrome Coronavirus 2 Infection' OR 'SARS Coronavirus 2 Infection' OR 'COVID-19 Virus Disease' OR 'COVID 19 Virus Disease' OR 'COVID-19 Virus Diseases' OR '2019-nCoV Infection' OR '2019 nCoV Infection' OR '2019-nCoV Infections' OR 'COVID19' OR 'COVID-19 Pandemic' OR 'COVID 19 Pandemic' OR 'COVID-19 Pandemics'
#3	MeSH descriptor: [Extracorporeal Membrane Oxygenation] explode all trees
#4	'Extracorporeal Membrane Oxygenations' OR 'ECLS Treatment' OR 'ECLS Treatments' OR 'Extracorporeal Life Support' OR 'Extracorporeal Life Supports' OR 'ECMO Treatment' OR 'ECMO Treatments' OR 'ECMO Extracorporeal Membrane Oxygenation' OR 'Venoarterial ECMO' OR 'Venoarterial ECMOs' OR 'Venoarterial Extracorporeal Membrane Oxygenation' OR 'Venovenous ECMO' OR 'Venovenous ECMOs' OR 'Venovenous Extracorporeal Membrane Oxygenation'
#5	MeSH descriptor: [Outcome] explode all trees
#6	'Prognos*' OR 'Surviv*' OR 'Mortalit*' OR 'Fatal*' OR 'Outcome'
#7	#1 OR #2
#8	#3 OR #4
#9	#5 OR #6
#10	#7 AND #8 AND #9

Table S3. Studies excluded (n=150) with reasons.

Studies excluded	Reasons
Correa, 2021 [1]	Without sufficient data
Dakroub, 2021 [2]	Without sufficient data
Guihaire, 2020 [3]	Without sufficient data
Hua, 2021 [4]	Without sufficient data
Karagiannidis, 2021 [5]	Without sufficient data
Lee, 2022 [6]	Without sufficient data
Levy, 2021 [7]	Without sufficient data
Li, 2021 [8]	Without sufficient data
Loforte, 2020 [9]	Without sufficient data
Mathilde, 2022 [10]	Without sufficient data
Moyon, 2022 [11]	Without sufficient data
Nguyen, 2021 [12]	Without sufficient data
Pans, 2022 [13]	Without sufficient data
Patel, 2022 [14]	Without sufficient data
Rieg, 2022 [15]	Without sufficient data
Rodrigues, 2021 [16]	Without sufficient data
Sterling, 2022 [17]	Without sufficient data
Supady, 2021 [18]	Without sufficient data
Whebell, 2022 [19]	Without sufficient data
Yang, 2022 [20]	Without sufficient data
Yankah, 2021 [21]	Without sufficient data
Yaqoob, 2021 [22]	Without sufficient data
Zappella, 2022 [23]	Without sufficient data
Giraud, 2021 [24]	Repeated population
Herrmann, 2020 [25]	Repeated population
Jacobs, 2021 [26]	Repeated population
Jacobs, 2020 [27]	Repeated population
Zha, 2021 [28]	Repeated population
Placeholder name,2020 [29]	Without full text
Placeholder name,2021 [30]	Without full text

Placeholder name,2022 [31]	Without full text
Bonilla, 2020 [32]	Without full text
Cavaliere, 2022 [33]	Without full text
Dave, 2020 [34]	Without full text
Duculan, 2022 [35]	Without full text
Fatima, 2022 [36]	Without full text
Gannon, 2022 [37]	Without full text
Griffee, 2020 [38]	Without full text
Hayanga, 2022 [39]	Without full text
Hernandez, 2022 [40]	Without full text
Hu, 2020 [41]	Without full text
Kumar, 2022 [42]	Without full text
Ling. 2022 [43]	Without full text
Martucci, 2021 [44]	Without full text
Nasirov, 2022 [45]	Without full text
Ohshimo, 2022 [46]	Without full text
Pavone, 2020 [47]	Without full text
Pirani, 2021 [48]	Without full text
Rajeswaran, 2022 [49]	Without full text
Remmington, 2022 [50]	Without full text
Tang, 2021 [51]	Without full text
Zaaqoq, 2022 [52]	Without full text
Ahmad, 2022[53]	Without appropriate comparison and intervention: non-invacive respiratory support time
Al-Yousif, 2022[54]	Without appropriate comparison and intervention: IMV and ECMO
Alfraij, 2021[55]	Without appropriate comparison and intervention: ICU survivors and non-survivors
Alhumaid, 2021[56]	Without appropriate comparison and intervention: ECMO and non-ECMO
Annie, 2022[57]	Without appropriate comparison and intervention: sex differences
Arabi, 2021[58]	Without appropriate comparison and intervention: without comparison
Benes, 2022[59]	Without appropriate comparison and intervention: ICU survival and non-survival
Bermea, 2021[60]	Without appropriate comparison and intervention: different inflammatory markers
Bissel, 2022[61]	Without appropriate comparison and intervention: without comparison,
Cain, 2021[62]	Without appropriate comparison and intervention: right ventricular assist device and ECMO
De Hesselle, 2022[63]	Without appropriate comparison and intervention: ECMO and other treatments
Dodd, 2022[64]	Without appropriate comparison and intervention: without comparison
Fanelli, 2021[65]	Without appropriate comparison and intervention: ECMO and non-ECMO
Fang, 2021[66]	Without appropriate comparison and intervention: weaned and non-weaned
Gjurasin, 2021[67]	Without appropriate comparison and intervention: influenza and COVID-19
Gresser, 2021[68]	Without appropriate comparison and intervention: without comparison
Heubner, 2022[69]	Without appropriate comparison and intervention: ECMO or

	not
Hu, 2020[70]	Without appropriate comparison and intervention: invasive respiratory support or not
Ippolito, 2022[71]	Without appropriate comparison and intervention: ECMO or not
Kim, 2021[72]	Without appropriate comparison and intervention: survival and non-survival
Klein, 2020[73]	Without appropriate comparison and intervention: IMV or not
Lee, 2020[74]	Without appropriate comparison and intervention: hospital survival and non-survival
Li, 2020[75]	Without appropriate comparison and intervention: hospital survival and non-survival
Li, 2021[76]	Without appropriate comparison and intervention: ECMO or not
Mang, 2022[77]	Without appropriate comparison and intervention: control cohort and awake cohort
Montrucchio, 2022[78]	Without appropriate comparison and intervention: survival and non-survival
Mustafa, 2021[79]	Without appropriate comparison and intervention: ECMO or maximum ventilation alone
Oliveira, 2021[80]	Without appropriate comparison and intervention: ICU survival and non-survival
Premraj, 2021[81]	Without appropriate comparison and intervention: ECMO or not
Reddy, 2021[82]	Without appropriate comparison and intervention: COVID-19 first wave and second wave
Richard, 2022[83]	Without appropriate comparison and intervention: ECMO or not
Schallner, 2022[84]	Without appropriate comparison and intervention: ECMO or not, COVID-19 or not
Smith, 2022[85]	Without appropriate comparison and intervention: different ECMO types
Taylor, 2022[86]	Without appropriate comparison and intervention: ECMO and no ECMO
Urner, 2022[87]	Without appropriate comparison and intervention: ECMO and conventional mechanical ventilation
Weatherill, 2022[88]	Without appropriate comparison and intervention: COVID-19 and non-COVID-19
Willers, 2021[89]	Without appropriate comparison and intervention: V-V ECMO and V-A ECMO
Besa, 2021 [90]	Without appropriate population: 90% COVID-19 patients
Bharat, 2021 [91]	Without appropriate population: after lung transplantation
Cavalcante, 2022 [92]	Without appropriate population: children
Di Nardo, 2022 [93]	Without appropriate population: children
Li, 2021 [94]	Without appropriate population: with mechanical circulatory support
Mc Fadyen, 2022 [95]	Without appropriate population: sildenafil use
Omar, 2022 [96]	Without appropriate population: non-COVID-19 patients
Voicu, 2021 [97]	Without appropriate population: corticosteroid treated
Worku, 2022 [98]	Without appropriate population: non-COVID-19 patients
Zakrajsek, 2022 [99]	Without appropriate population: asthma patients
Al Mutair, 2021 [100]	Without appropriate outcome: survival and non-survival characteristics
Alser, 2021 [101]	Without appropriate outcome: baseline review
Bergman, 2021 [102]	Without appropriate outcome: prone positioning before ECMO

	and other baseline characteristics
Blaize, 2022 [103]	Without appropriate outcome: candidemia
Brozzi, 2020 [104]	Without appropriate outcome: hemorrhagic complications
Cervantes-Arslanian, 2022 [105]	Without appropriate outcome: neurologic manifestation
Chandel, 2021 [106]	Without appropriate outcome: macro thrombosis
Chang, 2021 [107]	Without appropriate outcome: deep vein thrombosis
Cho, 2021 [108]	Without appropriate outcome: stroke
Desborough, 2020 [109]	Without appropriate outcome: venous thromboembolism
Durak, 2021 [110]	Without appropriate outcome: thromboembolic and bleeding events
Fragao-Marques, 2021 [111]	Without appropriate outcome: thromboembolic and bleeding events
Ghosn, 2021 [112]	Without appropriate outcome: acute kidney injury
Guo, 2021 [113]	Without appropriate outcome: anticoagulation
Koukaki, 2022 [114]	Without appropriate outcome: fungal infection
Leasure, 2021 [115]	Without appropriate outcome: intracerebral hemorrhage
Lesan, 2021 [116]	Without appropriate outcome: immunoglobulin-like receptor 2DS5
Mansour, 2022 [117]	Without appropriate outcome: immunoglobulin-like receptor 2DS5
Zaaqoq, 2021 [118]	Without appropriate outcome: inflammation and coagulation
Ahmadi, 2020 [119]	Case report
Al-Mumin, 2022 [120]	Case report
Barrantes, 2021 [121]	Case report
Bussolari, 2021 [122]	Case report
Chao, 2020 [123]	Case report
Chen, 2020 [124]	Case report
Elmenlliti, 2022 [125]	Case report
Granata, 2022 [126]	Case report
Gulmez, 2022 [127]	Case report
Hekimian, 2021 [128]	Case report
Herth, 2020 [129]	Case report
Hu, 2020 [130]	Case report
Huang, 2021 [131]	Case report
Huette, 2020 [132]	Case report
Ichiyanma, 2022 [133]	Case report
Janc, 2022 [134]	Case report
Kakar, 2021 [135]	Case report
Kaman, 2021 [136]	Case report
Kannapadi, 2021 [137]	Case report
Kucuk, 2022 [138]	Case report
Le Breton, 2020 [139]	Case report
Li, 2020 [140]	Case report
Nagaoka, 2021 [141]	Case report
Ponce, 2022 [142]	Case report
Rafiq, 2020 [143]	Case report
Sakai, 2022 [144]	Case report
Sen, 2021[145]	Case report
Sultan, 2020[146]	Case report
Xuan, 2020[147]	Case report
Yin, 2022[148]	Case report
Zhang, 2020[149]	Case report
Zhang, 2021[150]	Case report

Table S4. Quality assessment of included studies.

Author (Publication Year)	Newcastle-Ottawa Scale									
	Selection			Comparability			Outcome			Total
	a	b	c	d	e	f	g	h	i	
Akkanti, 2022	1	1	1	1	0	0	1	1	1	7
Alnababteh, 2021	1	1	1	1	0	0	1	0	1	6
Bergman, 2021	1	1	1	1	0	0	1	1	1	7
Beyl, 2022	1	1	1	1	1	1	1	1	1	9
Biancari, 2021	1	1	1	1	0	0	1	1	1	7
Blazoski, 2021	1	1	1	1	0	0	1	0	1	6
Braaten, 2022	1	0	1	1	0	0	1	1	1	6
Casabella, 2021	1	1	1	1	0	0	1	0	1	6
Charlton, 2021	1	1	1	1	0	0	1	0	1	6
Cheng, 2021	1	1	1	1	0	0	1	1	1	7
Daviet, 2021	1	0	1	1	1	1	1	1	1	8
Diaz, 2021	1	1	1	1	1	0	1	1	1	8
Dreier, 2021	1	1	1	1	0	0	1	1	1	7
Gannon, 2022	1	1	1	1	0	0	1	0	1	6
Hajage, 2022	1	1	1	1	0	0	1	1	1	7
Hall, 2022	1	1	1	1	1	1	1	1	1	9
Haroun, 2022	1	1	1	1	0	0	1	0	1	6
Hermann, 2022	1	1	1	1	1	1	1	0	1	8
Herrmann, 2022	1	1	1	1	1	1	1	0	1	8
Jacobs, 2022	1	1	1	1	0	0	1	0	1	6
Kunavarapu, 2021	1	1	1	1	1	1	1	0	1	8
Lai, 2021	1	1	1	1	0	0	1	0	1	6
Lang, 2021	1	1	1	1	0	0	1	1	1	7
Lebreton, 2021	1	1	1	1	1	1	1	1	1	9
Lee, 2022	1	1	1	1	0	0	1	0	1	6
Levy, 2022	1	1	1	1	0	0	1	0	1	6
Loforte, 2021	1	1	1	1	0	0	1	0	1	6
Maharaj, 2022	1	1	1	1	0	0	1	0	1	6
Mongero, 2021	1	1	1	1	0	0	1	0	1	6
Natanov, 2022	1	1	1	1	0	0	1	0	1	6
Nesseler, 2022	1	1	1	1	1	1	1	1	1	9
Olivier, 2021	1	1	1	1	0	0	1	0	1	6
Pacheco, 2020	1	1	0	1	0	0	1	0	1	5
Powell, 2022	1	1	1	1	0	0	1	0	1	6
Rabie, 2021	1	0	1	1	1	1	1	0	1	7
Raff, 2020	1	1	1	1	1	1	1	0	1	8
Rajajee, 2021	1	1	1	1	0	0	1	1	1	7
Riera, 2022	1	1	1	1	1	1	1	1	1	9
Saeed, 2022	1	1	1	1	1	1	1	1	1	9
Saeed, 2022	1	1	1	1	1	1	1	1	1	9
Schmidt, 2020	1	1	1	1	0	0	1	1	1	7
Schmidt, 2021	1	0	1	1	1	1	1	1	1	7
Shaefi, 2021	1	1	1	1	0	0	1	1	1	7
Supady, 2021	1	1	1	1	0	0	1	1	1	7
Takeuchi, 2022	1	0	1	1	0	0	1	0	1	5
Trejnowska, 2022	1	1	1	1	0	0	1	0	1	6
Varghese, 2021	1	1	0	1	0	0	1	0	1	5
Vigneshwar, 2022	1	1	1	1	0	0	1	0	1	6
Yang, 2020	1	1	1	1	0	0	1	0	1	6
Yaqoob, 2022	1	1	0	1	0	0	1	0	1	5
Yoshino, 2021	1	1	0	1	0	0	1	0	1	5
Zaaqoq, 2022	1	1	1	1	0	0	1	0	1	6
Zayat, 2021	1	1	1	1	0	0	1	0	1	6
Zhang, 2020	1	1	1	1	0	0	1	0	1	6

a. Representativeness of the exposed cohort. b. Selection of the non-exposed cohort. c. Ascertainment of exposure. d. Demonstration that outcome of interest was not present at start of study. e. Comparability of cohorts on the basis of the design or analysis (adjusted for age). f. Comparability of cohorts on the basis of the design or analysis (adjusted for any other factor). g.

Assessment of outcome. h. Was follow-up long enough for outcomes to occur (> 1 month). i. Adequacy of follow-up of cohorts.

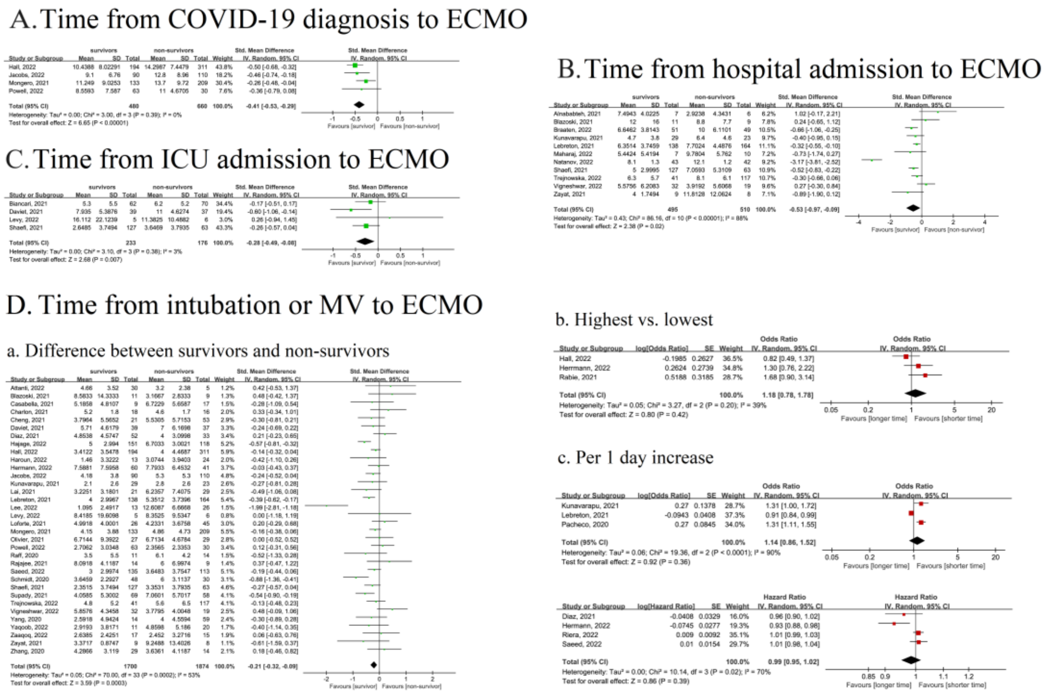

Figure S1. A. Forest plot showing the differences in time from COVID-19 diagnosis to ECMO between survivors and non-survivors in COVID-19 patients; B. Forest plot showing the differences in time from hospital admission to ECMO between survivors and non-survivors in COVID-19 patients; C. Forest plot showing the differences in time from ICU admission to ECMO between survivors and non-survivors in COVID-19 patients; D. Forest plot for the association between time from intubation or MV to ECMO and survival in COVID-19 patients. (a). Forest plot showing the differences in time between survivors and non-survivors in COVID-19 patients. (b). Forest plot for the association between time and survival, analyzed as category variables (highest vs. lowest). (c). Forest plot for the association between time and survival, analyzed as continuous variables (per one-day increase).

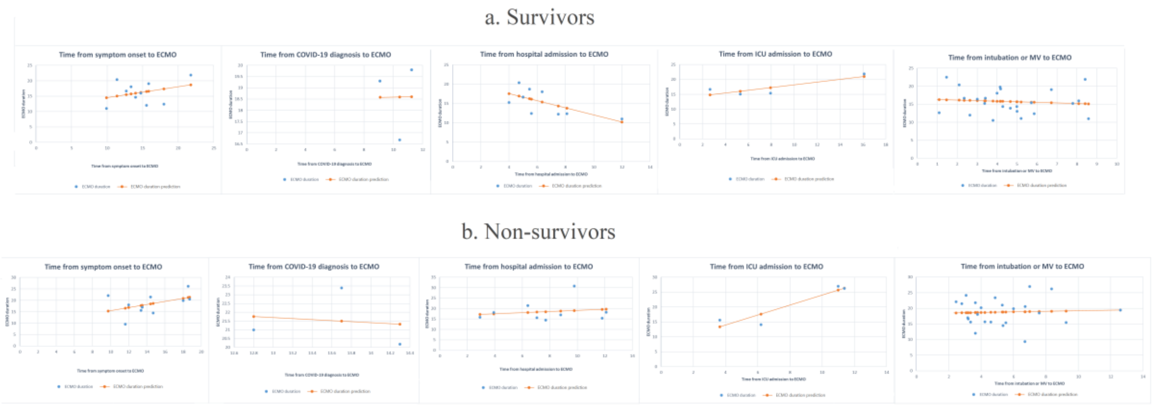

Figure S2. Regression analysis of pre-ECMO time and ECMO duration. a. Survivors; b. Non-survivors.

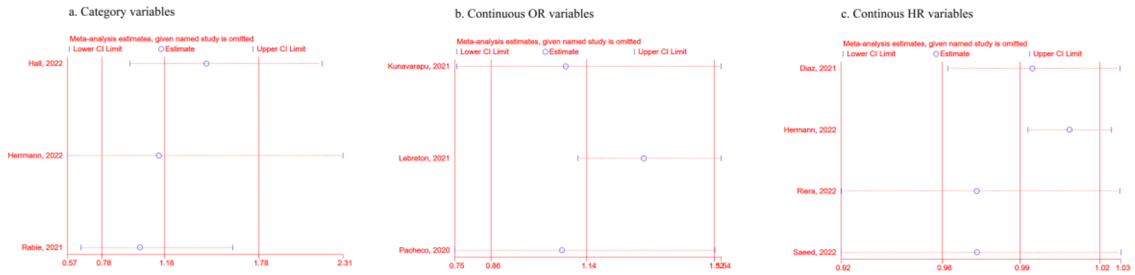

Figure S3. Sensitivity analysis of time from MV or intubation to ECMO difference in COVID-19 for mortality by omitting one study at once. a. Category variables; b. Continuous OR variables; c. Continuous HR variables.

Reference

1. Corrêa TD, Midega TD, Timenetsky KT, et al. Clinical characteristics and outcomes of COVID-19 patients admitted to the intensive care unit during the first year of the pandemic in Brazil: a single center retrospective cohort study. *Einstein (Sao Paulo)* 2021; 19: eAO6739. 2021/12/09. https://doi.org/10.31744/einstein_journal/2021AO6739.
2. Dakroub F, Fakhredine S, Yassine M, et al. A retrospective analysis of 902 hospitalized COVID-19 patients in Lebanon: clinical epidemiology and risk factors. *J Clin Virol Plus* 2021; 1: 100048. 2022/03/10. https://doi.org/10.1016/j.jcvp.2021.100048.
3. Guihaire J, Owyang CG, Madhok J, et al. Specific Considerations for Venovenous Extracorporeal Membrane Oxygenation during Coronavirus Disease 2019 Pandemic. *ASAIO Journal* 2020: 1069-1072. Article in Press. https://doi.org/10.1097/MAT.0000000000001251.
4. Hua J, Zhang X, Wang N, et al. Extracorporeal Membrane Oxygenation for COVID-19: Case Report of Nine Patients. *Frontiers in Medicine* 2021; 8. Article. https://doi.org/10.3389/fmed.2021.697338.
5. Karagiannidis C, Strassmann S, Merten M, et al. High In-Hospital Mortality Rate in Patients with COVID-19 Receiving Extracorporeal Membrane Oxygenation in Germany: A Critical Analysis. *Am J Respir Crit Care Med* 2021; 204: 991-994. 2021/07/21. https://doi.org/10.1164/rccm.202105-1145LE.
6. Lee JH, Ahn JS, Chung MJ, et al. Development and Validation of a Multimodal-Based Prognosis and Intervention Prediction Model for COVID-19 Patients in a Multicenter Cohort. *Sensors (Basel)* 2022; 22 2022/07/10. https://doi.org/10.3390/s22135007.
7. Levy D, Lebreton G, Pineton de Chambrun M, et al. Outcomes of Patients Denied Extracorporeal Membrane Oxygenation during the COVID-19 Pandemic in Greater Paris, France. *Am J Respir Crit Care Med* 2021; 204: 994-997. 2021/08/11. https://doi.org/10.1164/rccm.202105-1312LE.
8. Li X, Hu M, Zheng R, et al. Delayed Initiation of ECMO Is Associated With Poor Outcomes in Patients With Severe COVID-19: A Multicenter Retrospective Cohort Study. *Front Med (Lausanne)* 2021; 8: 716086. 2021/10/05. https://doi.org/10.3389/fmed.2021.716086.
9. Loforte A, Dal Checco E, Gliozzi G, et al. Veno-venous extracorporeal membrane oxygenation support in covid-19 respiratory distress syndrome: Initial experience. *ASAIO Journal* 2020: 734-738. Article in Press. https://doi.org/10.1097/MAT.0000000000001198.
10. Mathilde N, Bérénice P, Dorothée V, et al. Late venovenous extracorporeal membrane oxygenation in patients with acute respiratory distress syndrome due to SARS-CoV-2. *Annals of Intensive Care* 2022; 12. Conference Abstract. https://doi.org/10.1186/s13613-022-01016-6.
11. Moyon Q, Pineton de Chambrun M, Lebreton G, et al. Validation of survival prediction models for ECMO in Sars-CoV-2-related acute respiratory distress syndrome. *Crit Care* 2022; 26: 187. 2022/06/22. https://doi.org/10.1186/s13054-022-04039-4.
12. Nguyen NT, Sullivan B, Sagebin F, et al. Analysis of COVID-19 Patients With Acute Respiratory Distress Syndrome Managed With Extracorporeal Membrane Oxygenation at US Academic Centers. *Ann Surg* 2021; 274: 40-44. 2021/04/13. https://doi.org/10.1097/sla.0000000000004870.
13. Pans N, Vanherf J, Vandenbrande J, et al. Predictors of poor outcome in critically ill patients with COVID-19 pneumonia treated with extracorporeal membrane oxygenation. *Perfusion* 2022: 2676591221131487. Article in Press. https://doi.org/10.1177/02676591221131487.
14. Patel YJ, Stokes JW, Gannon WD, et al. Extracorporeal Membrane Oxygenation Circuits in Parallel for Refractory Hypoxemia in COVID-19: A Case Series. *Asaio j* 2022; 68: 1002-1009. 2022/05/05. https://doi.org/10.1097/mat.0000000000001706.
15. Rieg S, von Cube M, Kalbhenn J, et al. COVID-19 in-hospital mortality and mode of death in a dynamic and non-restricted tertiary care model in Germany. *PLoS One* 2020; 15: e0242127. 2020/11/13. https://doi.org/10.1371/journal.pone.0242127.
16. Rodrigues AB, Neves I, Gomes D, et al. Venovenous ECMO support in patients with SARS-COV-2 infection complicated with severe acute respiratory failure-a single centre cohort study. *Intensive Care Medicine Experimental* 2021; 9. Conference Abstract. https://doi.org/10.1186/s40635-021-00413-8.

17. Sterling R, Anderson B, Batchinsky A, et al. A Single Center Experience with Early Initiation of ECMO Prior to Mechanical Ventilation in COVID-19 Patients with Severe ARDS. *ASAIO Journal* 2022; 68: 42. Conference Abstract.
18. Supady A, Taccone FS, Lepper PM, et al. Survival after extracorporeal membrane oxygenation in severe COVID-19 ARDS: results from an international multicenter registry. *Crit Care* 2021; 25: 90. 2021/03/03. https://doi.org/10.1186/s13054-021-03486-9.
19. Whebell S, Zhang J, Lewis R, et al. Survival benefit of extracorporeal membrane oxygenation in severe COVID-19: a multi-centre-matched cohort study. *Intensive Care Medicine* 2022; 48: 467-478. Article. https://doi.org/10.1007/s00134-022-06645-w.
20. Yang X, Cai S, Luo Y, et al. Extracorporeal Membrane Oxygenation for Coronavirus Disease 2019-Induced Acute Respiratory Distress Syndrome: A Multicenter Descriptive Study. *Crit Care Med* 2020; 48: 1289-1295. 2020/05/20. https://doi.org/10.1097/ccm.0000000000004447.
21. Yankah CA, Trimlett R, Sandoval E, et al. COVID-19 Pulmonary Failure and Extracorporeal Membrane Oxygenation: First Experience from Three European Extracorporeal Membrane Oxygenation Centers. *Thoracic and Cardiovascular Surgeon* 2021; 69: 259-262. Article. https://doi.org/10.1055/s-0040-1719156.
22. Yaqoob H, Arshad A, Greenberg D, et al. EXTRACORPOREAL MEMBRANE OXYGENATION IN PATIENTS WITH COVID-19: A RETROSPECTIVE ANALYSIS AT A QUATERNARY CENTER. *Chest* 2021; 160: A1146-A1147. Conference Abstract. https://doi.org/10.1016/j.chest.2021.07.1049.
23. Zappella N, Dirani C, Lortat Jacob B, et al. Temporary ICUs during the COVID-19 pandemic first wave: description of the cohort at a French centre. *BMC Anesthesiol* 2022; 22: 310. 2022/10/04. https://doi.org/10.1186/s12871-022-01845-9.
24. Giraud R, Legouis D, Assouline B, et al. Timing of VV-ECMO therapy implementation influences prognosis of COVID-19 patients. *Physiol Rep* 2021; 9: e14715. 2021/02/03. https://doi.org/10.14814/phy2.14715.
25. Herrmann J, Adam EH, Notz Q, et al. COVID-19 Induced Acute Respiratory Distress Syndrome-A Multicenter Observational Study. *Front Med (Lausanne)* 2020; 7: 599533. 2021/01/05. https://doi.org/10.3389/fmed.2020.599533.
26. Jacobs JP, Stammers AH, Louis JS, et al. Multi-institutional Analysis of 100 Consecutive Patients with COVID-19 and Severe Pulmonary Compromise Treated with Extracorporeal Membrane Oxygenation: Outcomes and Trends Over Time. *Asaio j* 2021; 67: 496-502. 2021/04/27. https://doi.org/10.1097/mat.0000000000001434.
27. Jacobs JP, Stammers AH, St Louis J, et al. Extracorporeal Membrane Oxygenation in the Treatment of Severe Pulmonary and Cardiac Compromise in Coronavirus Disease 2019: Experience with 32 Patients. *Asaio j* 2020; 66: 722-730. 2020/04/23. https://doi.org/10.1097/mat.0000000000001185.
28. Zha L, Sobue T, Takeuchi T, et al. Characteristics and Survival of Intensive Care Unit Patients with Coronavirus Disease in Osaka, Japan: A Retrospective Observational Study. *J Clin Med* 2021; 10 2021/07/03. https://doi.org/10.3390/jcm10112477.
29. [Recommendations on extracorporeal life support for critically ill patients with novel coronavirus pneumonia]. *Zhonghua Jie He He Hu Xi Za Zhi* 2020; 43: 195-198. 2020/03/14. https://doi.org/10.3760/cma.j.issn.1001-0939.2020.03.011.
30. 37th Annual Children's National Symposium: ECMO and the Advanced Therapies forCardiovascular and Respiratory Failure. *ASAIO Journal* 2021; 67. Conference Review.
31. ECMO 2022 Annual Meeting Abstracts. *ASAIO Journal* 2022; 68. Conference Review.
32. Bonilla C, Riera J, Pacheco A, et al. Complications during ECMO support in patients with COVID-19: A prospective observational study. *ASAIO Journal* 2020; 66: 17. Conference Abstract.
33. Cavaliere N, Leong R and Maarouf O. The effect of renal dysfunction on mortality rates for patients on VVECMO for ARDS due to COVID-19 infection. *ASAIO Journal* 2022; 68: 24. Conference Abstract.
34. Dave S, Rabinowitz R, Shah A, et al. Outcomes of Venovenous Extracorporeal Membrane Oxygenation for Acute Respiratory Failure in Viral infections. *ASAIO Journal* 2020; 66: 24. Conference Abstract.
35. Duculan T and Zainab A. Prolonged ECMO Therapy Outcomes: What's Next? *ASAIO Journal* 2022; 68: 18. Conference Abstract.
36. Fatima S, Quindoy R, Zainab A, et al. A multidisciplinary effort to improve ECMO management during the COVID-19 pandemic. *Perfusion* 2022; 37: 45. Conference Abstract. https://doi.org/10.1177/02676591221089240.
37. Gannon WD, Stokes JW, Francois SA, et al. Association between Availability of Extracorporeal Membrane Oxygenation and Mortality in Patients with COVID-19 Eligible for Extracorporeal Membrane Oxygenation: A Natural Experiment. *Am J Respir Crit Care Med* 2022; 205: 1354-1357. 2022/02/26. https://doi.org/10.1164/rccm.202110-2399LE.
38. Griffee M. The COVID-19 Critical Care Consortium Global Study: Preliminary Summary of 183 Cases of ECMO. *ASAIO Journal* 2020; 66: 17. Conference Abstract.
39. Hayanga JA, Song T, Durham L, et al. Early initiation of hemoadsorption reduces the need for organ support in critically ill COVID-19 patients on ECMO: A post-hoc analysis from the CytoSorb Therapy in COVID-19 (CTC) Registry. *ASAIO Journal* 2022; 68: 22. Conference Abstract.
40. Hernández L, Jiménez-Rodríguez GM, Espinosa-Gonzalez P, et al. Outcomes in Extracorporeal Membrane Oxygenation (ECMO) singlecenter experience in Latin America. *ASAIO Journal* 2022; 68: 15. Conference Abstract.
41. Hu B and Peng Z. The authors reply. *Crit Care Med* 2020; 48: e1358. 2020/12/02. https://doi.org/10.1097/ccm.0000000000004656.
42. Kumar N, Burns R, Crown L, et al. ECMO Duration for COVID-19 Patients - Does it Matter? *ASAIO Journal* 2022; 68: 25. Conference Abstract.
43. Ling R, Ramanathan K, Shen L, et al. Immunomodulators in patients receiving extracorporeal membrane oxygenation for COVID-19: a propensity-score weighted analysis of the ELSO registry. *ASAIO Journal* 2022; 68: 26. Conference Abstract.

44. Martucci G, Słomka A, Lebowitz SE, et al. COVID-19 and Extracorporeal Membrane Oxygenation. 2021, p. 173-195.
45. Nasirov T, Gao B, Jones J, et al. Rapid Expansion of VV-ECMO Support during the COVID-19 Pandemic: Experiences from a Community Hospital. *ASAIO Journal* 2022; 68: 66. Conference Abstract.
46. Ohshimo S, Liu K, Ogura T, et al. Impact of high/low-volume hospitals of ECMO before the COVID-19 pandemic era on ECMO outcomes in patients with fatal COVID-19. *ASAIO Journal* 2022; 68: 27. Conference Abstract.
47. Pavone N, Burzotta F and Massetti M. Extracorporeal membrane oxygenation for COVID-19: effective weapon or futile effort? *Minerva Cardioangiol* 2020; 68: 365-367. 2020/11/07. https://doi.org/10.23736/s0026-4725.20.05377-3.
48. Pirani T, Patel S, Loveridge R, et al. Veno-venous extracorporeal membrane oxygenation (V-V ECMO) for Coronavirus Disease 2019 (COVID-19) related refractory respiratory failure in liver transplantation (LT) recipients: A single centre experience. *Transplantation* 2021; 105: 171-172. Conference Abstract. https://doi.org/10.1097/01.tp.0000789500.50801.c7.
49. Rajeswaran N, Aw TC, Garfield B, et al. Life-threatening bradycardias in COVID-19 patients whilst supported on venous-venous ECMO. *ASAIO Journal* 2022; 68: 6. Conference Abstract.
50. Remmington C, Rose L, Hanks F, et al. A single centre retrospective observational study comparing the sedative requirements of patients with COVID-19 requiring ECMO compared to a historical control. *Perfusion* 2022; 37: 50. Conference Abstract. https://doi.org/10.1177/02676591221089240.
51. Tang X, Pu L, Zhang JY, et al. [Comparison of extracorporeal membrane oxygenation applicated in critical patients with COVID-19 and novel influenza A (H1N1) virus pneumonia]. *Zhonghua Yi Xue Za Zhi* 2021; 101: 579-585. 2021/03/06. https://doi.org/10.3760/cma.j.cn112137-20201007-02776.
52. Zaaqoq A, Griffee M, Kelly TL, et al. Cerebrovascular complications for COVID-19 patients supported by veno-venous extracorporeal membrane oxygenation. *Perfusion* 2022; 37: 42-44. Conference Abstract. https://doi.org/10.1177/02676591221089240.
53. Ahmad Q, Green A, Chandel A, et al. Impact of Noninvasive Respiratory Support in Patients With COVID-19 Requiring V-V ECMO. *Asaio j* 2022; 68: 171-177. 2022/01/29. https://doi.org/10.1097/mat.0000000000001626.
54. Al-Yousif N, Komanduri S, Qurashi H, et al. Radiographic Assessment of Lung Edema (RALE) Scores are Highly Reproducible and Prognostic of Clinical Outcomes for Inpatients with COVID-19. *medRxiv* 2022 2022/06/24. https://doi.org/10.1101/2022.06.10.22276249.
55. Alfraij A, Bin Alamir AA, Al-Otaibi AM, et al. Characteristics and outcomes of coronavirus disease 2019 (COVID-19) in critically ill pediatric patients admitted to the intensive care unit: A multicenter retrospective cohort study. *J Infect Public Health* 2021; 14: 193-200. 2021/01/25. https://doi.org/10.1016/j.jiph.2020.12.010.
56. Alhumaid S, Al Mutair A, Alghazal HA, et al. Extracorporeal membrane oxygenation support for SARS-CoV-2: a multi-centered, prospective, observational study in critically ill 92 patients in Saudi Arabia. *Eur J Med Res* 2021; 26: 141. 2021/12/11. https://doi.org/10.1186/s40001-021-00618-3.
57. Annie FH, Dave S, Nanjundappa A, et al. Sex Differences of Extracorporeal Membrane Oxygenation Distribution Among SARS-COV-2 Cases. *Angiology* 2022; 73: 283-284. 2021/10/09. https://doi.org/10.1177/00033197211049748.
58. Arabi J, Duewell B, Juul J, et al. Evaluation of anticoagulation in COVID-19 patients requiring extracorporeal membrane oxygenation. *Critical Care Medicine* 2021; 49: 78. Conference Abstract. https://doi.org/10.1097/01.ccm.0000726632.84003.06.
59. Benes J, Jankowski M, Szułdrzynski K, et al. SepsEast Registry indicates high mortality associated with COVID-19 caused acute respiratory failure in Central-Eastern European intensive care units. *Sci Rep* 2022; 12: 14906. 2022/09/02. https://doi.org/10.1038/s41598-022-18991-2.
60. Bermea RS, Raz Y, Sertic F, et al. Increased Intracranial Hemorrhage Amid Elevated Inflammatory Markers in Those With COVID-19 Supported With Extracorporeal Membrane Oxygenation. *Shock (Augusta, Ga)* 2021; 56: 206-214. Article. https://doi.org/10.1097/SHK.0000000000001730.
61. Bissell BD, Gabbard T, Sheridan EA, et al. Evaluation of Bivalirudin as the Primary Anticoagulant in Patients Receiving Extracorporeal Membrane Oxygenation for SARS-CoV-2-Associated Acute Respiratory Failure. *Ann Pharmacother* 2022; 56: 387-392. 2021/07/30. https://doi.org/10.1177/10600280211036151.
62. Cain MT, Smith NJ, Barash M, et al. Extracorporeal Membrane Oxygenation with Right Ventricular Assist Device for COVID-19 ARDS. *J Surg Res* 2021; 264: 81-89. 2021/04/01. https://doi.org/10.1016/j.jss.2021.03.017.
63. de Hesselle ML, Borgmann S, Rieg S, et al. Invasiveness of Ventilation Therapy Is Associated to Prevalence of Secondary Bacterial and Fungal Infections in Critically Ill COVID-19 Patients. *J Clin Med* 2022; 11 2022/09/10. https://doi.org/10.3390/jcm11175239.
64. Dodd K, Crabbe S, Schoenrade N, et al. Outcomes following prolonged venovenous extracorporeal membrane oxygenation in COVID-19. *Critical Care Medicine* 2022; 50: 125. Conference Abstract. https://doi.org/10.1097/01.ccm.0000807444.57044.d3.
65. Fanelli V, Montrucchio G, Sales G, et al. Effects of Steroids and Tocilizumab on the Immune Response Profile of Patients with COVID-19-Associated ARDS Requiring or Not Veno-Venous Extracorporeal Membrane Oxygenation. *Membranes (Basel)* 2021; 11 2021/08/27. https://doi.org/10.3390/membranes11080603.
66. Fang J, Li R, Chen Y, et al. Extracorporeal Membrane Oxygenation Therapy for Critically Ill Coronavirus Disease 2019 Patients in Wuhan, China: A Retrospective Multicenter Cohort Study. *Curr Med Sci* 2021; 41: 1-13. 2021/02/15. https://doi.org/10.1007/s11596-021-2311-8.

67. Gjurašin B, Santini M, Krajinović V, et al. A retrospective comparison between influenza and COVID-19-associated ARDS in a Croatian tertiary care center. *Wien Klin Wochenschr* 2021; 133: 406-411. 2020/11/21. https://doi.org/10.1007/s00508-020-01759-x.
68. Gresser E, Reich J, Sabel BO, et al. Risk stratification for ecmo requirement in covid-19 icu patients using quantitative imaging features in ct scans on admission. *Diagnostics* 2021; 11: NA. Article. https://doi.org/10.3390/diagnostics11061029.
69. Heubner L, Greiner M, Vicent O, et al. Predictive ability of viscoelastic testing using ClotPro® for short-term outcome in patients with severe Covid-19 ARDS with or without ECMO therapy: a retrospective study. *Thromb J* 2022; 20: 48. 2022/08/30. https://doi.org/10.1186/s12959-022-00403-0.
70. Hu HT, Xu S, Wang J, et al. Respiratory Support in Severely or Critically Ill ICU Patients With COVID-19 in Wuhan, China. *Curr Med Sci* 2020; 40: 636-641. 2020/08/09. https://doi.org/10.1007/s11596-020-2227-8.
71. Ippolito A, Urban H, Ghoroghi K, et al. Prevalence of acute neurological complications and pathological neuroimaging findings in critically ill COVID-19 patients with and without VV-ECMO treatment. *Sci Rep* 2022; 12: 17423. 2022/10/20. https://doi.org/10.1038/s41598-022-21475-y.
72. Kim EJ, Lee YH, Park JS, et al. Clinical features and prognostic factors of critically ill patients with COVID-19 in Daegu, South Korea: A multi-center retrospective study. *Medicine* 2021; 100: e24437. Article. https://doi.org/10.1097/MD.0000000000024437.
73. Klein SJ, Bellmann R, Dejaco H, et al. Structured ICU resource management in a pandemic is associated with favorable outcome in critically ill COVID‑19 patients. *Wien Klin Wochenschr* 2020; 132: 653-663. 2020/11/11. https://doi.org/10.1007/s00508-020-01764-0.
74. Lee SG, Park GU, Moon YR, et al. Clinical Characteristics and Risk Factors for Fatality and Severity in Patients with Coronavirus Disease in Korea: A Nationwide Population-Based Retrospective Study Using the Korean Health Insurance Review and Assessment Service (HIRA) Database. *Int J Environ Res Public Health* 2020; 17 2020/11/22. https://doi.org/10.3390/ijerph17228559.
75. Li J, Xu G, Yu H, et al. Clinical Characteristics and Outcomes of 74 Patients With Severe or Critical COVID-19. *Am J Med Sci* 2020; 360: 229-235. 2020/07/13. https://doi.org/10.1016/j.amjms.2020.05.040.
76. Li S, Xiong J, Du Z, et al. Extracorporeal membrane oxygenation (ECMO) for critically ill patients with coronavirus disease 2019 (COVID-19): A retrospective cohort study. *J Card Surg* 2021; 36: 3554-3560. 2021/07/23. https://doi.org/10.1111/jocs.15833.
77. Mang S, Reyher C, Mutlak H, et al. Awake Extracorporeal Membrane Oxygenation for COVID-19-induced Acute Respiratory Distress Syndrome. *Am J Respir Crit Care Med* 2022; 205: 847-851. 2022/01/20. https://doi.org/10.1164/rccm.202105-1189LE.
78. Montrucchio G, Corcione S, Lupia T, et al. The Burden of Carbapenem-Resistant Acinetobacter baumannii in ICU COVID-19 Patients: A Regional Experience. *J Clin Med* 2022; 11 2022/09/10. https://doi.org/10.3390/jcm11175208.
79. Mustafa AK, Joshi DJ, Alexander PJ, et al. Comparative Propensity Matched Outcomes in Severe COVID-19 Respiratory Failure-Extracorporeal Membrane Oxygenation or Maximum Ventilation Alone. *Ann Surg* 2021; 274: e388-e394. 2021/10/08. https://doi.org/10.1097/sla.0000000000005187.
80. Oliveira E, Parikh A, Lopez-Ruiz A, et al. ICU outcomes and survival in patients with severe COVID-19 in the largest health care system in central Florida. *PLoS One* 2021; 16: e0249038. 2021/03/26. https://doi.org/10.1371/journal.pone.0249038.
81. Premraj L, Zaaqoq A, Barnett A, et al. Stroke in ECMO patients with COVID-19: An analysis of the COVID?19 Critical Care Consortium (CCCC) international, multicentre observational study. *Intensive Care Medicine Experimental* 2021; 9. Conference Abstract. https://doi.org/10.1186/s40635-021-00413-8.
82. Reddy R, Dovidio J, Baram M, et al. OUTCOMES OF EXTRACORPOREAL MEMBRANE OXYGENATION IN ARDS DUE TO COVID-19: COMPARISON OF THE FIRST AND THE SECOND WAVE. *Chest* 2021; 160: A1065. Conference Abstract. https://doi.org/10.1016/j.chest.2021.07.985.
83. Richard JC, Sigaud F, Gaillet M, et al. Response to PEEP in COVID-19 ARDS patients with and without extracorporeal membrane oxygenation. A multicenter case-control computed tomography study. *Crit Care* 2022; 26: 195. 2022/07/03. https://doi.org/10.1186/s13054-022-04076-z.
84. Schallner N, Lieberum J, Kalbhenn J, et al. Intensive care unit resources and patient-centred outcomes in severe COVID-19: a prospective single-centre economic evaluation. *Anaesthesia* 2022 2022/08/31. https://doi.org/10.1111/anae.15844.
85. Smith NJ, Park S, Zundel MT, et al. Extracorporeal membrane oxygenation for COVID-19: An evolving experience through multiple waves. *Artif Organs* 2022 2022/08/13. https://doi.org/10.1111/aor.14381.
86. Taylor LJ, Jolley SE, Ramani C, et al. Early posthospitalization recovery after extracorporeal membrane oxygenation in survivors of COVID-19. *J Thorac Cardiovasc Surg* 2022 2022/04/19. https://doi.org/10.1016/j.jtcvs.2021.11.099.
87. Urner M, Barnett AG, Bassi GL, et al. Venovenous extracorporeal membrane oxygenation in patients with acute covid-19 associated respiratory failure: comparative effectiveness study. *The BMJ* 2022; 377. Article. https://doi.org/10.1136/bmj-2021-068723.
88. Weatherill A, Laffan M, Gasper M, et al. Impact of Thrombosis and Bleeding in Patients with Severe COVID-19 versus Other Viral Pneumonias in the Context of Extracorporeal Membrane Oxygenation. *Semin Thromb Hemost* 2022; 48: 118-123. 2021/08/14. https://doi.org/10.1055/s-0041-1732371.
89. Willers A, Swol J, Buscher H, et al. Elso registry analysis-longitudinal trends in bleeding complications on extracorporeal life support (ECLS) over the past twenty years. *ASAIO Journal* 2021; 67: 14. Conference Abstract.

90. Besa S, Fuenzalida R, Lagos C, et al. Implementation and outcomes of a successful extracorporeal membrane oxygenation mobile retrieval service in chile during the sars-cov-2 pandemic. *ASAIO Journal* 2021; 67: 51. Conference Abstract.
91. Bharat A, Machuca TN, Querrey M, et al. Early outcomes after lung transplantation for severe COVID-19: a series of the first consecutive cases from four countries. *Lancet Respir Med* 2021; 9: 487-497. 2021/04/04. https://doi.org/10.1016/s2213-2600(21)00077-1.
92. Cavalcante C, de Oliveira Teles AC, Maia ICL, et al. Extracorporeal membrane oxygenation support in children with severe coronavirus disease-2019: A case series. *Lancet Reg Health Am* 2022; 11: 100260. 2022/05/17. https://doi.org/10.1016/j.lana.2022.100260.
93. Di Nardo M, De Piero ME, Hoskote A, et al. Extracorporeal membrane oxygenation in children with COVID-19 and PIMS-TS during the second and third wave. *Lancet Child Adolesc Health* 2022; 6: e14-e15. 2022/03/07. https://doi.org/10.1016/s2352-4642(22)00065-7.
94. Li R, Hu S, Chen P, et al. Saving critically ill COVID-19 patients with mechanical circulatory support. *Ann Transl Med* 2021; 9: 1221. 2021/09/18. https://doi.org/10.21037/atm-20-5169.
95. McFadyen C, Garfield B, Mancio J, et al. Use of sildenafil in patients with severe COVID-19 pneumonitis. *Br J Anaesth* 2022; 129: e18-e21. 2022/05/15. https://doi.org/10.1016/j.bja.2022.04.004.
96. Omar AS, Labib A, Hanoura SE, et al. Impact of Extracorporeal Membrane Oxygenation Service on Burnout Development in Eight Intensive Care Units. A National Cross-Sectional Study. *J Cardiothorac Vasc Anesth* 2022; 36: 2891-2899. 2022/03/19. https://doi.org/10.1053/j.jvca.2022.02.018.
97. Voicu S, Goury A, Lacoste-Palasset T, et al. Dismal Survival in COVID-19 Patients Requiring ECMO as Rescue Therapy after Corticosteroid Failure. *J Pers Med* 2021; 11 2021/11/28. https://doi.org/10.3390/jpm11111238.
98. Worku ET, Yeung F, Anstey C, et al. The impact of reduction in intensity of mechanical ventilation upon venovenous ECMO initiation on radiographically assessed lung edema scores: A retrospective observational study. *Front Med (Lausanne)* 2022; 9: 1005192. 2022/10/08. https://doi.org/10.3389/fmed.2022.1005192.
99. Zakrajsek JK, Min SJ, Ho PM, et al. Extracorporeal Membrane Oxygenation (ECMO) for Refractory Asthma Exacerbations with Respiratory Failure. *Chest* 2022 2022/10/04. https://doi.org/10.1016/j.chest.2022.09.029.
100. Al Mutair A, Elhazmi A, Alhumaid S, et al. Examining the Clinical Prognosis of Critically Ill Patients with COVID-19 Admitted to Intensive Care Units: A Nationwide Saudi Study. *Medicina (Kaunas)* 2021; 57 2021/09/29. https://doi.org/10.3390/medicina57090878.
101. Alser O, Mokhtari A, Naar L, et al. Multisystem outcomes and predictors of mortality in critically ill patients with COVID-19: Demographics and disease acuity matter more than comorbidities or treatment modalities. *J Trauma Acute Care Surg* 2021; 90: 880-890. 2021/04/24. https://doi.org/10.1097/ta.0000000000003085.
102. Bergman ZR, Wothe JK, Alwan FS, et al. The Use of Venovenous Extracorporeal Membrane Oxygenation in COVID-19 Infection: One Region's Comprehensive Experience. *ASAIO Journal* 2021; 67: 503-510. Article. https://doi.org/10.1097/MAT.0000000000001403.
103. Blaize M, Raoelina A, Kornblum D, et al. Occurrence of Candidemia in Patients with COVID-19 Admitted to Five ICUs in France. *J Fungi (Basel)* 2022; 8 2022/07/28. https://doi.org/10.3390/jof8070678.
104. Brozzi N, Hernandez-Montfort J, Cudemus G, et al. High Rate of Hemorrhagic Complications in COVID-19 Patients Requiring ECMO Support in Spite of Conservative Anticoagulation Strategies. *ASAIO Journal* 2020; 66: 10. Conference Abstract.
105. Cervantes-Arslanian AM, Venkata C, Anand P, et al. Neurologic Manifestations of Severe Acute Respiratory Syndrome Coronavirus 2 Infection in Hospitalized Patients During the First Year of the COVID-19 Pandemic. *Crit Care Explor* 2022; 4: e0686. 2022/05/03. https://doi.org/10.1097/cce.0000000000000686.
106. Chandel A, Patolia S, Looby M, et al. Association of D-dimer and Fibrinogen With Hypercoagulability in COVID-19 Requiring Extracorporeal Membrane Oxygenation. *J Intensive Care Med* 2021; 36: 689-695. 2021/03/02. https://doi.org/10.1177/0885066621997039.
107. Chang H, Rockman CB, Jacobowitz GR, et al. Deep vein thrombosis in hospitalized patients with coronavirus disease 2019. *J Vasc Surg Venous Lymphat Disord* 2021; 9: 597-604. 2020/10/12. https://doi.org/10.1016/j.jvsv.2020.09.010.
108. Cho SM, Premraj L, Fanning J, et al. Ischemic and Hemorrhagic Stroke Among Critically Ill Patients With Coronavirus Disease 2019: An International Multicenter Coronavirus Disease 2019 Critical Care Consortium Study. *Crit Care Med* 2021; 49: e1223-e1233. 2021/07/17. https://doi.org/10.1097/ccm.0000000000005209.
109. Desborough MJR, Doyle AJ, Griffiths A, et al. Image-proven thromboembolism in patients with severe COVID-19 in a tertiary critical care unit in the United Kingdom. *Thromb Res* 2020; 193: 1-4. 2020/06/03. https://doi.org/10.1016/j.thromres.2020.05.049.
110. Durak K, Kersten A, Grottke O, et al. Thromboembolic and Bleeding Events in COVID-19 Patients receiving Extracorporeal Membrane Oxygenation. *Thoracic and Cardiovascular Surgeon* 2021; 69: 526-536. Article. https://doi.org/10.1055/s-0041-1725180.
111. Fragao-Marques M, Barroso I, Loureiro H, et al. Inflammatory Response Assessment in Patients with COVID-19 Under Extracorporeal Membrane Oxygenation Support. *Clin Lab* 2021; 67 2021/05/13. https://doi.org/10.7754/Clin.Lab.2020.200916.
112. Ghosn M, Attallah N, Badr M, et al. Severe Acute Kidney Injury in Critically Ill Patients with COVID-19 Admitted to ICU: Incidence, Risk Factors, and Outcomes. *J Clin Med* 2021; 10 2021/04/04. https://doi.org/10.3390/jcm10061217.

113. Guo Z, Sun L, Li B, et al. Anticoagulation Management in Severe Coronavirus Disease 2019 Patients on Extracorporeal Membrane Oxygenation. *Journal of Cardiothoracic and Vascular Anesthesia* 2021; 35: 389-397. Article. https://doi.org/10.1053/j.jvca.2020.08.067.
114. Koukaki E, Rovina N, Tzannis K, et al. Fungal Infections in the ICU during the COVID-19 Era: Descriptive and Comparative Analysis of 178 Patients. *J Fungi (Basel)* 2022; 8 2022/08/27. https://doi.org/10.3390/jof8080881.
115. Leasure AC, Khan YM, Iyer R, et al. Intracerebral Hemorrhage in Patients With COVID-19: An Analysis From the COVID-19 Cardiovascular Disease Registry. *Stroke* 2021; 52: e321-e323. 2021/06/05. https://doi.org/10.1161/strokeaha.121.034215.
116. Lesan V, Bewarder M, Metz C, et al. Killer immunoglobulin-like receptor 2DS5 is associated with recovery from coronavirus disease 2019. *Intensive Care Med Exp* 2021; 9: 45. 2021/09/04. https://doi.org/10.1186/s40635-021-00409-4.
117. Mansour A, Flecher E, Schmidt M, et al. Bleeding and thrombotic events in patients with severe COVID-19 supported with extracorporeal membrane oxygenation: a nationwide cohort study. *Intensive Care Med* 2022; 48: 1039-1052. 2022/07/14. https://doi.org/10.1007/s00134-022-06794-y.
118. Zaaqoq A, Sallam T, Merley C, et al. The interplay of inflammation and coagulation in covid-19 patients supported by extracorporeal membrane oxygenation. *ASAIO Journal* 2021; 67: 12. Conference Abstract.
119. Ahmadi ZH, Jahangirifard A, Farzanegan B, et al. Extracorporeal membrane oxygenation and COVID-19: The causes of failure. *Journal of Cardiac Surgery* 2020; 35: 2838-2843. Article. https://doi.org/10.1111/jocs.14867.
120. Al-Mumin A, Tarakemeh H, Buabbas S, et al. Liberation from Mechanical Ventilation Before Decannulation from Venovenous Extracorporeal Life Support in Severe COVID-19 Acute Respiratory Distress Syndrome. *ASAIO journal (American Society for Artificial Internal Organs : 1992)* 2022. Article in Press. https://doi.org/10.1097/MAT.0000000000001806.
121. Barrantes JH, Ortoleva J, O'Neil ER, et al. Successful Treatment of Pregnant and Postpartum Women With Severe COVID-19 Associated Acute Respiratory Distress Syndrome With Extracorporeal Membrane Oxygenation. *Asaio j* 2021; 67: 132-136. 2020/11/25. https://doi.org/10.1097/mat.0000000000001357.
122. Bussolari C, Palumbo D, Fominsky E, et al. Case Report: Nintedaninb May Accelerate Lung Recovery in Critical Coronavirus Disease 2019. *Front Med (Lausanne)* 2021; 8: 766486. 2021/11/16. https://doi.org/10.3389/fmed.2021.766486.
123. Chao CJ, DeValeria PA, Sen A, et al. Reversible cardiac dysfunction in severe COVID-19 infection, mechanisms and case report. *Echocardiography* 2020; 37: 1465-1469. 2020/08/29. https://doi.org/10.1111/echo.14807.
124. Chen JY, Qiao K, Liu F, et al. Lung transplantation as therapeutic option in acute respiratory distress syndrome for coronavirus disease 2019-related pulmonary fibrosis. *Chin Med J (Engl)* 2020; 133: 1390-1396. 2020/04/07. https://doi.org/10.1097/cm9.0000000000000839.
125. Elmelliti H, Mutkule DP, Imran M, et al. Bleeding Hazard of Percutaneous Tracheostomy in COVID-19 Patients Supported With Venovenous Extracorporeal Membrane Oxygenation: A Case Series. *Journal of Cardiothoracic and Vascular Anesthesia* 2022. Article in Press. https://doi.org/10.1053/j.jvca.2022.09.084.
126. Granata A, Martucci G, Rizzo GEM, et al. Combined endoscopical treatments for tracheo-esophageal fistula developed during V-V ECMO for severe COVID-19: A case series. *Artif Organs* 2022; 46: 506-508. 2021/11/11. https://doi.org/10.1111/aor.14101.
127. Gulmez DD, Yilmaz ET, Karamustafa M, et al. Extracorporeal membrane oxygenation experience in COVID-19 pandemic: Report of two cases. *North Clin Istanb* 2022; 9: 275-278. 2020/11/24. https://doi.org/10.14744/nci.2020.86094.
128. Hékimian G, Kerneis M, Zeitouni M, et al. Coronavirus Disease 2019 Acute Myocarditis and Multisystem Inflammatory Syndrome in Adult Intensive and Cardiac Care Units. *Chest* 2021; 159: 657-662. 2020/09/11. https://doi.org/10.1016/j.chest.2020.08.2099.
129. Herth FJF, Sakoulas G and Haddad F. Use of Intravenous Immunoglobulin (Prevagen or Octagam) for the Treatment of COVID-19: Retrospective Case Series. *Respiration* 2020; 99: 1145-1153. 2020/12/15. https://doi.org/10.1159/000511376.
130. Hu L, Peng K, Huang X, et al. A novel strategy sequentially linking mechanical cardiopulmonary resuscitation with extracorporeal cardiopulmonary resuscitation optimizes prognosis of refractory cardiac arrest: an illustrative case series. *Eur J Med Res* 2022; 27: 77. 2022/06/02. https://doi.org/10.1186/s40001-022-00711-1.
131. Huang S, Xia H, Wu Z, et al. Clinical data of early COVID-19 cases receiving extracorporeal membrane oxygenation in Wuhan, China. *J Clin Anesth* 2021; 68: 110044. 2020/11/02. https://doi.org/10.1016/j.jclinane.2020.110044.
132. Huette P, Beyls C, Guilbart M, et al. Extracorporeal membrane oxygenation for respiratory failure in COVID-19 patients: outcome and time-course of clinical and biological parameters. *Can J Anaesth* 2020; 67: 1486-1488. 2020/06/03. https://doi.org/10.1007/s12630-020-01727-z.
133. Ichiyama T, Komatsu M, Wada Y, et al. Report of a combination of remdesivir, intravenous methylprednisolone pulse, and tocilizumab for severe coronavirus disease: 20-case series at a single institution. *Respir Investig* 2022; 60: 604-606. 2022/05/03. https://doi.org/10.1016/j.resinv.2022.04.001.
134. Janc J, Łysenko L, Lewandowska O, et al. A Successful Outcome of Veno-Venous Extracorporeal Membrane Oxygenation in Obese Patients with Respiratory Failure in the Course of COVID-19: A Report of Two Cases. *International Journal of Environmental Research and Public Health* 2022; 19. Article. https://doi.org/10.3390/ijerph19052761.
135. Kakar V, North A, Bajwa G, et al. Long Runs and Higher Incidence of Bleeding Complications in COVID-19 Patients Requiring Venovenous Extracorporeal Membrane Oxygenation: A Case Series from the United Arab Emirates. *Indian J Crit Care Med* 2021; 25: 1452-1458. 2022/01/15. https://doi.org/10.5005/jp-journals-10071-24054.

136. Kaman K, Azmy V, Chichra A, et al. Cytokine profiles in severe SARS-CoV-2 infection requiring extracorporeal membrane oxygenation support. *Respir Med Case Rep* 2021; 33: 101376. 2021/03/09. https://doi.org/10.1016/j.rmcr.2021.101376.
137. Kannapadi NV, Jami M, Premraj L, et al. Neurologic Injury in Patients With COVID-19 Who Receive VV-ECMO Therapy: A Cohort Study. *J Cardiothorac Vasc Anesth* 2021; 35: 3456-3461. 2021/06/14. https://doi.org/10.1053/j.jvca.2021.05.017.
138. Kucuk AO, Küçük MP, Ayçiçek O, et al. Extracorporeal membrane oxygenation experiences during COVID-19 pandemic, third wave with younger patients: A retrospective observational study. *Turkish Journal of Emergency Medicine* 2022; 22: 36-43. Article. https://doi.org/10.4103/2452-2473.336106.
139. Le Breton C, Besset S, Freita-Ramos S, et al. Extracorporeal membrane oxygenation for refractory COVID-19 acute respiratory distress syndrome. *J Crit Care* 2020; 60: 10-12. 2020/07/31. https://doi.org/10.1016/j.jcrc.2020.07.013.
140. Li X, Guo Z, Li B, et al. Extracorporeal Membrane Oxygenation for Coronavirus Disease 2019 in Shanghai, China. *Asaio j* 2020; 66: 475-481. 2020/04/04. https://doi.org/10.1097/mat.0000000000001172.
141. Nagaoka E, Arai H, Ugawa T, et al. Efficacy of multidisciplinary team approach with extracorporeal membrane oxygenation for COVID-19 in a low volume ECMO center. *Artif Organs* 2021; 45: 1061-1067. 2021/03/04. https://doi.org/10.1111/aor.13947.
142. Ponce D, de Carvalho RLR, Pires MC, et al. Extracorporeal membrane oxygenation outcomes in COVID-19 patients: Case series from the Brazilian COVID-19 Registry. *Artificial Organs* 2022; 46: 964-971. Article. https://doi.org/10.1111/aor.14136.
143. Rafiq MU, Valchanov K, Vuylsteke A, et al. Regional extracorporeal membrane oxygenation retrieval service during the severe acute respiratory syndrome coronavirus 2 (SARS-CoV-2) pandemic: an interdisciplinary team approach to maintain service provision despite increased demand. *Eur J Cardiothorac Surg* 2020; 58: 875-880. 2020/09/24. https://doi.org/10.1093/ejcts/ezaa327.
144. Sakai T, Hoshino C, Nakano M, et al. Rehabilitation Characteristics of Acute-stage COVID-19 Survivors Managed with Extracorporeal Membrane Oxygenation in the Intensive Care Unit. *Prog Rehabil Med* 2022; 7: 20220015. 2022/04/19. https://doi.org/10.2490/prm.20220015.
145. Sen A, Blakeman S, DeValeria PA, et al. Practical Considerations for and Outcomes of Interfacility ECMO Transfer of Patients With COVID-19 During a Pandemic: Mayo Clinic Experience. *Mayo Clin Proc Innov Qual Outcomes* 2021; 5: 525-531. 2021/03/10. https://doi.org/10.1016/j.mayocpiqo.2021.02.004.
146. Sultan I, Habertheuer A, Usman AA, et al. The role of extracorporeal life support for patients with COVID-19: Preliminary results from a statewide experience. *Journal of Cardiac Surgery* 2020; 35: 1410-1413. Article. https://doi.org/10.1111/jocs.14583.
147. Xuan W, Chen C, Jiang X, et al. Clinical characteristics and outcomes of five critical COVID-19 patients treated with extracorporeal membrane oxygenation in Leishenshan Hospital in Wuhan. *J Clin Anesth* 2020; 67: 110033. 2020/09/10. https://doi.org/10.1016/j.jclinane.2020.110033.
148. Yin O, Richley M, Hadaya J, et al. Extracorporeal membrane oxygenation in pregnancy: a bridge to delivery and pulmonary recovery for COVID-19-related severe respiratory failure. *Am J Obstet Gynecol* 2022; 226: 571-576.e575. 2021/12/20. https://doi.org/10.1016/j.ajog.2021.12.024.
149. Zhang B, Liu S, Tan T, et al. Treatment With Convalescent Plasma for Critically Ill Patients With Severe Acute Respiratory Syndrome Coronavirus 2 Infection. *Chest* 2020; 158: e9-e13. 2020/04/04. https://doi.org/10.1016/j.chest.2020.03.039.
150. Zhang JC and Li T. Delayed retroperitoneal hemorrhage during extracorporeal membrane oxygenation in COVID-19 patients: A case report and literature review. *World Journal of Clinical Cases* 2021; 9: 5203-5210. Article. https://doi.org/10.12998/wjcc.v9.i19.5203.
